# Post-Quantum Revocable Linkable Ring Signature Scheme Based on SPHINCS+ for V2G Scenarios

**DOI:** 10.3390/s26030754

**Published:** 2026-01-23

**Authors:** Shuanggen Liu, Ya Nan Du, Xu An Wang, Xinyue Hu, Hui En Su

**Affiliations:** 1 School of Cyberspace Security, Xi’an University of Posts and Telecommunications, Xi’an 710121, China; liushuanggen201@xupt.edu.cn (S.L.); 15326880233@163.com (Y.N.D.); huxinyue0326@stu.xupt.edu.cn (X.H.); suhuien123@stu.xupt.edu.cn (H.E.S.); 2 Key Laboratory of Network and Information Security, Engineering University of People’s Armed Police, Xi’an 710086, China

**Keywords:** V2G networks, linkable ring signature, revocability, SPHINCS+

## Abstract

As a core support for the integration of new energy and smart grids, Vehicle-to-Grid (V2G) networks face a core contradiction between user privacy protection and transaction security traceability—a dilemma that is further exacerbated by issues such as the quantum computing vulnerability of traditional cryptography, cumbersome key management in stateful ring signatures, and conflicts between revocation mechanisms and privacy protection. To address these problems, this paper proposes a post-quantum revocable linkable ring signature scheme based on SPHINCS+, with the following core innovations: First, the scheme seamlessly integrates the pure hash-based architecture of SPHINCS+ with a stateless design, incorporating WOTS+, FORS, and XMSS technologies, which inherently resists quantum attacks and eliminates the need to track signature states, thus completely resolving the state management dilemma of traditional stateful schemes; second, the scheme introduces an innovative “real signature + pseudo-signature polynomially indistinguishable” mechanism, and by calibrating the authentication path structure and hash distribution of pseudo-signatures (satisfying the *Kolmogorov–Smirnov* test with D≤0.05), it ensures signer anonymity and mitigates the potential risk of distinguishable pseudo-signatures; third, the scheme designs a KEK (Key Encryption Key)-sharded collaborative revocation mechanism, encrypting and storing the (I,pk,RID) mapping table in fragmented form, with KEK split into $KEK_{1}$ (held by the Trusted Authority, TA) and $KEK_{2}$ (held by the regulatory node), with collaborative decryption by both parties required to locate malicious users, thereby resolving the core conflict of privacy leakage in traditional revocation mechanisms; fourth, the scheme generates forward-secure linkable tags based on one-way private key updates and one-time random factors, ensuring that past transactions cannot be traced even if the current private key is compromised; and fifth, the scheme adopts hash commitments instead of complex cryptographic commitments, simplifying computations while efficiently binding transaction amounts to signers—an approach consistent with the pure hash-based design philosophy of SPHINCS+. Security analysis demonstrates that the scheme satisfies the following six core properties: post-quantum security, unforgeability, anonymity, linkability, unframeability, and forward secrecy, thereby providing technical support for secure and anonymous payments in V2G networks in the quantum era.

## 1. Introduction

**Figure 1:**
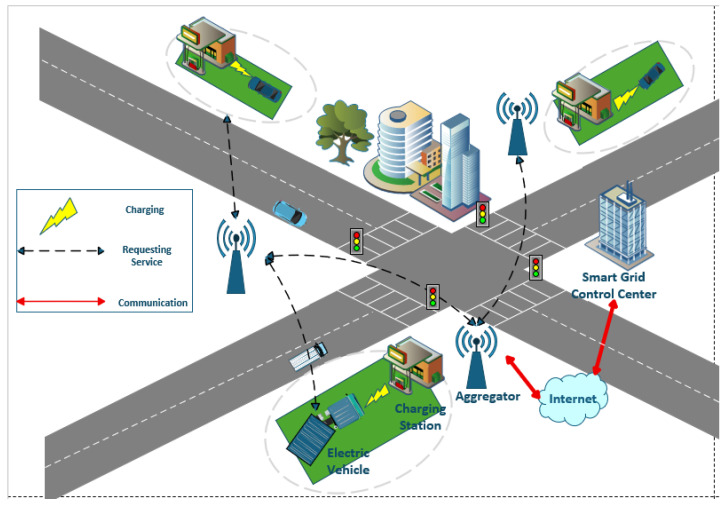
V2G network architecture.

The long-term rise in gasoline prices and the hazards of global warming have propelled Electric Vehicles (EVs) to become a global research and attention focus [1]. Boasting advantages such as environmental friendliness, high energy efficiency, and long driving range per charge, EVs have broad market prospects. It is predicted that the global market size of intelligent connected vehicles will reach trillions of US dollars by 2035. As a key component of Smart Grids (SGs), Vehicle-to-Grid (V2G) networks represent a core development trend for EVs [2,3,4]. Their typical architecture consists of the following five core components: EVs, Charging Stations (CSs), aggregators, Smart Grid Control Centers (SGCCs), and communication networks (as illustrated in Figure 1).

EVs upload information such as identity identifiers, battery status, and charging records to CSs. Aggregators then summarize and verify the stored data (from CSs) to monitor EV operating states before exchanging data with SGCCs via wired or wireless communication networks. SGCCs undertake multiple management responsibilities, including calculating total electricity prices based on charging and discharging demands and dispatching the entire V2G network [5]. The two-way power interaction between EVs and SGs generates a large volume of payment-related information [6], but during this interaction, attackers may target CSs or Local Aggregators to steal users’ charging records and other sensitive data. Such information—including identity details, living habits, geographical locations, and movement trajectories—could be maliciously exploited to facilitate violent crimes such as robberies and thefts, thereby endangering the personal safety of EV owners [7]. Thus, V2G networks urgently require a reliable payment scheme that balances privacy protection, transaction traceability, quantum attack resistance, and large-scale dynamic access to adapt to large-scale EV charging and discharging transactions.

Ring signatures [8], as a well-established digital signature technology, can realize identity authentication while providing users with anonymity protection. In a ring signature mechanism, the signer randomly selects public keys of multiple ring members to form a temporary group, then generates a signature by combining these public keys, their own private key, and random numbers, so verifiers can confirm that the signature originates from within the group but cannot identify the specific signer. The full anonymity and unforgeability of ring signatures have garnered widespread attention, with scholars exploring numerous application scenarios in vehicular networks in recent years [9,10]. However, the unconditional anonymity of ring signatures is overly absolute for certain scenarios such as electronic voting and electronic cash, and to address this, Liu et al. proposed the Linkable Spontaneous Anonymous Group Signature (LSAG) [11], a linkable ring signature (LRS) protocol that can identify whether two signatures are generated by the same private key while verifiers still cannot know the signer’s real identity. Unlike the strong unconditional anonymity of ring signatures, LRS offers linkable anonymity—an attribute that safeguards privacy in cryptocurrency applications while effectively mitigating double-spending attacks.

Nevertheless, existing LRS schemes suffer from significant flaws that hinder their practical application in V2G networks. Most existing LRS schemes rely on traditional cryptographic primitives and, thus, fail to resist quantum computing threats [12]—Shor’s algorithm can solve number-theoretic problems (e.g., discrete logarithms and integer factorization) in polynomial time, which poses unavoidable security risks in the quantum era. Many schemes adopt stateful mechanisms, which require continuous tracking of signature-related states (e.g., signature counts and private key indices) to ensure security. This not only increases key management complexity, but also fails to meet the requirements of V2G networks for dynamic access and long-term operation with a large fleet of EVs. The pseudo-signatures in traditional schemes differ from real signatures in structure or hash distribution, making them easily distinguishable, thus undermining anonymity. Revocation mechanisms in existing schemes either lack practicality or rely on centralized nodes to maintain complete mapping tables, leading to privacy leakage risks. Additionally, linkable tags in most schemes lack forward secrecy, leaving the risk of long-term tracking if private keys are leaked.

Existing privacy-preserving schemes for V2G networks primarily focus on general cryptographic attacks (e.g., forgery and anonymity violation) in their adversary models, yet fail to account for scenario-specific threats arising in practical V2G deployments. These unaddressed threats include the following: (1) malicious Charging Stations (CSs) tampering with transaction data (e.g., falsifying electricity consumption records); (2) malicious Local Aggregators (LAGs) forging signature verification results to collude with malicious EVs; (3) man-in-the-middle attacks targeting communication links between EVs and LAGs/BSs (e.g., altering transaction amounts or order timestamps); (4) side-channel attacks exploiting power consumption or timing differences of on-board terminals to leak private keys; and (5) collusion between malicious EVs and third parties to evade Trusted Authority (TA)-led revocation mechanisms. Such scenario-specific threats are rooted in the actual operational logic of V2G entities (e.g., LAGs’ mandate to verify signatures and forward transactions, as well as CSs’ responsibility to record charging data), thereby directly undermining the practical applicability of existing schemes. It is, therefore, imperative to refine the adversary model by explicitly incorporating these V2G-specific risks, ensuring that the security of the proposed scheme aligns with real-world deployment requirements.

Based on this, this paper proposes a SPHINCS+-based [13] linkable ring signature scheme, aiming to construct an anonymous payment mechanism with post-quantum security, high efficiency, and practicality for V2G networks. The core innovation of the proposed scheme resides in the seamless integration of SPHINCS+’s “pure hash-based” architecture, stateless design, and LRS’s privacy–traceability balancing mechanism, thereby systematically addressing the five key limitations of existing schemes. By leveraging WOTS+ one-time signatures [14], FORS hash-based signatures [15], and XMSS hierarchical tree structures [16], the scheme achieves stateless signature generation and verification—where signature generation depends solely on the private key seed and current transaction information, with no need to record historical state data. This fundamentally resolves the state management challenges of traditional schemes. It adopts a “structure calibration + hash distribution calibration” strategy for pseudo-signatures, where pseudo-signatures and real signatures use identical SPHINCS+ architecture parameters (16 FORS subtrees, 10 XMSS authentication path lengths) and pass the Kolmogorov–Smirnov test (D≤0.05) to ensure polynomially indistinguishable hash distributions, addressing the core risk of distinguishable pseudo-signatures. A Key Encryption Key (KEK)-sharded storage mechanism ($KEK=KEK_{1}⊕KEK_{2}$) is designed, with the TA and regulatory node each holding a key fragment; collaborative decryption is required to locate malicious users, resolving the conflict between revocation and privacy protection in traditional mechanisms. Forward-secure linkable tags are generated based on one-way private key updates and one-time random factors, ensuring that past transactions cannot be traced even if the current private key is leaked, eliminating the risk of long-term tracking. Hash commitments are used instead of complex cryptographic commitments, simplifying computations while efficiently binding transaction amounts to signers, which is consistent with SPHINCS+’s pure hash-based design philosophy and reduces the computational overhead of on-board terminals.

### 1.1. Related Work

In the research field of Vehicle-to-Grid (V2G) networks, the potential threats of quantum computing have become increasingly prominent, making post-quantum security a core consideration in privacy protection research. Currently, relevant studies primarily focus on the following three key directions: anonymous authentication, identity/location privacy protection, and privacy-preserving payment mechanisms. However, “how to efficiently achieve these goals in a post-quantum environment” remains a critical issue awaiting breakthroughs, as existing achievements generally lack sufficient adaptation to post-quantum security requirements.

Although existing V2G privacy-preserving schemes have achieved partial security objectives in specific scenarios, none of them fully address the risk of quantum attacks, and some exhibit obvious performance shortcomings. The P^2^ scheme [17], the first privacy-preserving V2G solution proposed by Yang et al., constructs a reward system and privacy-preserving communication functions through identity-based restrictive partially blind signatures. Nevertheless, it suffers from the flaw that generated certificates are vulnerable to forgery, and its traditional cryptographic foundation is inadequate to resist quantum attacks. Subsequently, Wang et al. [18] optimized this scheme by integrating bilinear pairing technology with the same type of blind signature technology. While remedying certain security vulnerabilities, the optimized scheme incurs significant computational and communication overheads and still fails to incorporate post-quantum defense mechanisms. Various privacy-preserving authentication protocols proposed in studies such as [19,20,21] focus on identity and location privacy protection in V2G networks but similarly lack systematic design for post-quantum security. To address privacy leakage in interactions between Electric Vehicles (EVs) and Smart Grids (SGs) in V2G networks, Liu et al. [9] proposed the EMULRS efficient multi-layer linkable ring signature scheme, which reduces overhead and optimizes performance with a logarithmic signature size. However, due to the absence of post-quantum cryptographic technologies, it cannot withstand quantum computing attacks. Additionally, while research in [22] systematically summarized privacy protection issues and corresponding solutions in V2G networks, it rarely addressed the security challenges of the post-quantum era, making it difficult to meet the long-term security needs of V2G systems.

Notably, the frequent exchange of payment information between EVs and SGs is prone to leaking sensitive data such as identity and location, a risk that quantum computing will further exacerbate. In recent years, although researchers have proposed various anonymous payment mechanisms to enhance privacy protection in V2G scenarios, gaps remain in their post-quantum security. Liu et al. [23] strengthened vehicle location privacy protection through an anonymous payment system but did not integrate post-quantum cryptographic technologies; Au et al. [24] enhanced the payment protocol, building on [23], by integrating BBS+ signaturesand zero-knowledge proof techniques, enabling both location privacy protection and stolen vehicle tracking. However, the protocol’s resistance to quantum attacks remains unvalidated, casting doubt on its security. Most of these studies focus on specific domains and have not yet formed a comprehensive, scalable framework suitable for practical scenarios and meeting post-quantum security requirements. Further exploration is needed to integrate post-quantum cryptography with the scalability of dynamic large-scale networks. Given the inherent anonymity advantages of ring signatures, combining them with post-quantum cryptography for V2G applications holds significant practical value, and relevant research urgently requires advancement.

As a matter of fact, comprehensive research into post-quantum ring signature technology has moved into a fast-track stage. Investigations carried out by academics like Xie and Wang have explicitly indicated that conventional digital signature mechanisms show notable susceptibility when confronted with quantum computing threats. In contrast, ring signatures, by dint of their distinctive technical attributes, have exhibited remarkable application merits across diverse blockchain scenarios—encompassing secure medical data sharing, in-vehicle network communications, and other domains [8]. Research groups headed by Chatterjee and Chung have concentrated on in-depth inquiries into the security of post-quantum ring signatures, re-evaluating the security thresholds of classical signature and ring signature technologies under quantum circumstances. They have also developed two short signature protocols, which have been validated and implemented in the quantum random oracle model and the standard model, respectively [25]. In recent pertinent publications, researchers have further expanded the technical modalities of post-quantum ring signatures, introducing innovative technical frameworks such as linkable ring signatures, threshold signatures, and identity-based post-quantum ring signatures. They have also conducted systematic analyses regarding the post-quantum security features of these architectures [26,27,28]. Correspondingly, an exhaustive overview of the theoretical framework and practical implementations of linkable ring signatures is provided in [29], carrying out in-depth comparisons of various technical approaches for anonymization design and linkability realization. Nevertheless, this study bears obvious shortcomings: it lacks targeted analyses for specific scenarios like the Internet of Things (IoT), and fails to fully take into account the adaptability issues of resource-limited settings, as well as the potential of quantum-resistant computation.

Focusing on the research orientations of the National Institute of Standards and Technology (NIST) in the realms of post-quantum algorithms and ring signature validation, certain scholars have put forward a blockchain-based post-quantum authentication protocol. This protocol integrates anonymity, traceability, and verifiability, efficiently fending off quantum attacks while striking a dynamic equilibrium between security safeguards and privacy protection. Moreover, it has successfully undergone feasibility validation in the in-vehicle network context [10]. The in-vehicle network scenario itself confronts severe security and privacy challenges stemming from device heterogeneity and frequent data exchanges. Blockchain technology, with its traits of decentralization, immutability, and distributed consensus, offers dependable technical backing for security protection in this field [30,31,32].

In particular, within the segmented scenarios of in-vehicle networks, the research on relevant security protocols has continued to deepen. To address privacy and anonymity in UAV-aided VANETs, Reference [33] proposed a specialized identity authentication and signature scheme tailored for Unmanned Aerial Vehicle (UAV)-aided Vehicular Ad Hoc Networks (VANETs), with an emphasis on boosting the anonymity of network communications and the capability of user privacy protection. Reference [34] developed a distributed message authentication scheme integrated with a reputation system, achieving message validity verification through dynamic assessment of node credit scores.This significantly enhances the in-vehicle network’s capacity to resist malicious assaults and data counterfeiting. The authenticated key agreement protocol for in-vehicle networks in intelligent transportation systems, proposed in Reference [35], proceeds from two aspects—strengthening identity authentication and optimizing key exchange—thus effectively warding off typical security hazards such as eavesdropping, tampering, and man-in-the-middle attacks.

Although the aforementioned studies have addressed numerous critical security issues in in-vehicle networks, they generally suffer from over-specificity to particular scenarios and lack a comprehensive, scalable framework suitable for practical implementation. More importantly, the integration of post-quantum cryptography with the scalability of dynamic large-scale networks has not been thoroughly explored, which provides a clear direction for subsequent research.

To establish a post-quantum security assurance system for data transmission in in-vehicle network and V2G environments, researchers have explored a variety of technical routes. For instance, Reference [36] suggested the adoption of lattice-based post-quantum digital signature technologies. However, this scheme remains merely at the theoretical level and does not involve practical design combined with specific application scenarios such as V2G. Reference [37], on the other hand, integrated the ring signature scheme based on lattice hard problems into the in-vehicle environment. Yet, the research failed to provide a detailed demonstration regarding the quantum attack resistance of the scheme, resulting in insufficient security evaluation.

Notably, reducing the energy consumption of blockchain technology in V2G network applications has also emerged as one of the current research focuses [38]. Some studies have developed energy-saving solutions by optimizing the calculation method of Merkle tree root nodes, proposed Merkle tree construction approaches that comply with industry standards, and confirmed the energy efficiency advantages of the scheme through experiments. The Merkle tree accumulator algorithm proposed by Derler and Ramacher in Reference [39] adopts a lightweight design that relies solely on hash functions and symmetric primitive languages. It possesses reliable quantum attack resistance and provides complete operational procedures and definition criteria. However, the engineering application specifics of this algorithm and its integrated utilization in practical scenarios such as V2G still require further in-depth investigation. Furthermore, the simultaneous satisfaction of the demand for anonymous transactions, the requirement for tracing malicious activities, and the need for post-quantum security among vehicle nodes in V2G networks has not yet been fully addressed.

Against this backdrop, linkable ring signature (LRS) [29], as a cryptographic primitive integrating anonymity and traceability, provides a new technical path for breaking through the aforementioned problems. Its core advantages lie in the following two aspects: on the one hand, the ring signature mechanism can ensure the identity anonymity of vehicle nodes in V2G networks, avoiding direct association between transaction information and real identities; on the other hand, “linkability” supports the traceability of multiple transactions from the same node, providing technical support for accountability for malicious activities. If a linkable ring signature scheme is constructed based on post-quantum cryptography (PQC), it can not only inherit the lightweight design to adapt to the resource-constrained characteristics of V2G networks, but also further enhance the system’s quantum attack resistance, remedying the deficiencies of existing Merkle tree-related schemes in the synergistic design of identity privacy protection and traceability. Therefore, combining linkable ring signatures with post-quantum cryptography and exploring their specific application modes and optimization strategies in V2G networks has become an important direction to address current research pain points.

Researchers have explored various linkable ring signature technologies. Reference [40] combined blockchain with the Internet of Vehicles (IoV), designed a linkable ring signature (LRS) based on the short integer solution (SIS) lattice assumption, and applied it to cold-chain logistics systems to achieve distributed data management, privacy protection, and transaction traceability. However, this scheme is only suitable for specific scenarios with insufficient versatility, has limited room for optimizing key size and time overhead, and its robustness against quantum attacks has not been further enhanced. Reference [41], based on the discrete logarithm assumption, converted 1-out-of-n ring signatures into t-out-of-n linkable threshold ring signatures (LTRSs) through sliding window transformation (SWT), and optimized the RingCT protocol to reduce the communication overhead of multi-input account transactions. Nevertheless, this protocol lacks post-quantum security, cannot resist quantum algorithm attacks, and has weak flexibility in threshold adjustment and signature scalability. Reference [42] proposed a certificateless multimode ring signcryption scheme (EMMCRS), integrating three modes (fully anonymous, linkable anonymous, and revocable anonymous). Based on elliptic curve cryptography, this scheme is suitable for Vehicular Ad Hoc Networks (VANETs) and supports batch verification, but suffers from low efficiency in switching anonymous modes, does not support the flexible addition or removal of dynamic ring members, and has high transmission latency in multi-node communication scenarios.

Reference [10] constructed an anonymous, traceable, and linkable authentication scheme for intelligent vehicle transportation systems based on NIST post-quantum winning algorithms (digital signatures and KEM), post-quantum linkable ring signatures, and consortium blockchain (Hyperledger Fabric). This scheme is equipped with a key exchange mechanism to support efficient encryption and decryption in P2P communication and broadcast scenarios. However, it does not adopt a pure hash-based architecture, so signature and verification efficiency is significantly affected by ring size; moreover, its non-stateless design increases key management complexity, resulting in limited ability to adapt to large-scale high-frequency transaction scenarios. Both Reference [43] and Reference [44] were based on lattice cryptography-related assumptions (the former on the SIS assumption, the latter on the module short integer solution (M-SIS) lattice assumption). They, respectively, designed an efficient linkable ring signature (LLRS) with a forward-secure enhanced version (FS-LLRS), and a logarithmic-scale signature scheme supporting anonymity and quantum attack resistance. The former is suitable for cloud-assisted electronic medical record sharing, while the latter is applied to electronic voting systems to prevent duplicate voting. However, both have high computational and communication overheads, their signature and verification efficiency are affected by ring size (linear growth for the former, logarithmic growth for the latter), and they lack flexibility in adapting to resource-constrained devices. Additionally, the latter relies on the random oracle model rather than the standard model and does not achieve a stateless design.

References [45,46,47] were all based on traditional cryptographic assumptions (bilinear groups with BDH assumption, elliptic curve discrete logarithm problem (ECDLP), and bilinear pairings with CDHP/DDHP assumptions). They, respectively, constructed an anonymous IoT data sharing scheme, a new PKI framework, and a blockchain–cloud–edge collaborative anonymous IoT data search and secure sharing scheme, realizing core requirements such as privacy protection and accountability. However, all these schemes lack post-quantum security; their signature and verification efficiency grow linearly with ring size, they have high key management complexity, and weak ability to adapt to large-scale high-frequency transaction scenarios. Reference [48] was based on the Ring-LWE assumption and lattice cryptography, integrating ring signcryption and non-interactive zero-knowledge proof (NIZKP) technology to build an anonymous linkable scheme suitable for VANET location services, ensuring vehicle query privacy, service provider data privacy, and anonymous payment with identity authentication. Nevertheless, the scheme has high computational and communication overheads, its signature and verification efficiency grow linearly with ring size, and its non-stateless design increases key management complexity.

Reference [49] was based on the SIS and LWE lattice assumptions, integrating non-interactive witness-indistinguishable (NIWI) proofs, strongly unforgeable one-time signatures (OTSs), and pseudorandom functions (PRFs) to construct a highly secure linkable ring signature scheme under the standard model, suitable for privacy protection in cryptocurrencies. However, the scheme’s signature and verification efficiency grow linearly with ring size, it has large key and signature sizes, and it does not achieve a stateless design. The HRPACS proposed in Reference [50] integrates blockchain, linkable ring signatures, homomorphic encryption, and an introducer mechanism, adopting a two-phase routing strategy and Blockchain-Tree structure to build a decentralized, anti-network-blocking non-end-to-end anonymous communication system. However, this system’s signature verification efficiency is affected by ring size and autonomous domain division, resulting in insufficient flexibility in adapting to high-frequency large-scale scenarios. The Lk-TRS designed in Reference [51] is based on bilinear groups with DL/DDH assumptions, integrating pseudorandom functions, accumulators, and signature proof of knowledge (SPK). It supports dynamic ring members, multi-account binding, and k-times signature tracing, suitable for fine-grained accountability in blockchain scenarios. However, its efficiency grows linearly with the k value, its non-stateless design increases key management complexity, and it has a weak ability to adapt to resource-constrained devices.

### 1.2. Contributions

To address the core drawbacks of existing schemes and the unique requirements of V2G network-specific scenarios, this paper proposes a post-quantum revocable linkable ring signature scheme based on SPHINCS+, with the following key contributions:

First, this paper, for the first time, deeply integrates the pure hash-based architecture of SPHINCS+ with linkable ring signatures. Leveraging WOTS+, FORS, and XMSS technologies, the scheme inherently resists quantum computing attacks and eliminates the need for signature state tracking. Signature generation depends solely on the private key seed and current transaction information, fundamentally solving the key management dilemma confronting traditional stateful schemes in large-scale dynamic access scenarios of V2G networks.

Secondly, an innovative dual strategy of “structure calibration + hash distribution calibration” is proposed to guarantee the polynomial indistinguishability of pseudo-signatures. Pseudo-signatures and real signatures adopt consistent SPHINCS+ architecture parameters (16 FORS subtrees, 10-layered XMSS authentication paths) and satisfy the *Kolmogorov–Smirnov* test (D≤0.05) to ensure indistinguishable hash distributions. This completely addresses the core risk of anonymity compromise caused by distinguishable pseudo-signatures in traditional schemes.

Third, a privacy-friendly collaborative revocation mechanism is constructed based on KEK (Key Encryption Key)-sharded storage and two-party collaborative decryption ($KEK=KEK_{1}⊕KEK_{2}$). The Trusted Authority (TA) and the regulatory node each hold a key fragment, and collaborative decryption by both parties is required to locate malicious users. This mechanism not only enables traceability of illegal activities, but also avoids privacy leakage caused by traditional centralized revocation, achieving a balance between anonymity protection and compliant supervision.

Fourth, forward secrecy for linkable tags is realized through one-way private key updates and one-time random factors. Even if the current private key is compromised, attackers cannot trace past transaction records, eliminating the risk of long-term tracking, thus adapting to the long-term security requirements of V2G network transactions.

Fifth, hash commitments are adopted instead of complex cryptographic commitments to achieve lightweight adaptation for resource-constrained scenarios. The scheme reuses the native hash functions of SPHINCS+ (e.g., SHA-256) without introducing complex primitives such as elliptic curves. While efficiently binding transaction amounts to signers, this design significantly reduces computational and storage overheads. Combined with hierarchical verification logic, the scheme is applicable to resource-constrained devices such as onboard terminals and charging stations, meeting the requirements of high-frequency transactions in V2G networks.

These contributions specifically address the key limitations of existing schemes in terms of quantum resistance, state management, anonymity, revocation mechanisms, and resource adaptability, providing a technically rigorous and practically viable solution for secure and anonymous payments in V2G networks in the quantum era.

### 1.3. Structure

This paper is structured as follows: First, Section 2 introduces the relevant preliminaries, including linkable ring signatures, WOTS+, FORS, and SPHINCS+, laying a theoretical foundation for the subsequent scheme design. Second, Section 3 elaborates on the design principles and detailed algorithm workflow of the proposed SPHINCS+-based linkable ring signature scheme, as well as its specific application scenarios in V2G networks. Third, Section 4 and Section 5, respectively, conduct comprehensive security analysis and quantitative performance analysis to verify the scheme’s feasibility and superiority. Finally, Section 6 concludes the whole paper and puts forward prospective future research directions.

## 2. Preliminaries

### 2.1. Linkable and Revocable Ring Signature

Ring-based signatures constitute a cryptographic signature framework proposed by Rivest, Tauman, and Shamir in 2001. This framework allows a signer to select ring members from a set of public keys and generate a signature by combining their own private key, the public keys of other ring members, and a random number, without disclosing the signer’s actual identity. The verifier can confirm that the signature originates from a member within the ring but cannot determine the specific identity of the signer.

As an augmented variant of ring signatures, the linkable ring signature is a digital signature mechanism that integrates both anonymity and linkability. It inherits the core logic of signing “in the name of a ring”, which conceals the precise identity of the signer within the ring. Furthermore, it introduces a “linking tag” to achieve a key functional upgrade: without exposing the signer’s identity, it enables determining whether two signatures are generated by the same signer via this tag.

The key parameters associated with the linkable and revocable ring signature (LRRS) are listed in Table 1.

**Definition** **1.** *(Linkable and Revocable Ring Signature). A linkable and revocable ring-based signature mechanism consists of the following six fundamental algorithms:*

*Parameter Generation: Setup(λ)→pp, which takes a security parameter λ as an input and outputs the system public parameters pp (including RID generation rules).*

*Key Generation: $\mathrm{KeyGen}\left(\lambda \right)\to \left(pk_{i},sk_{i},RID_{i}\right)$, which takes a security parameter λ as an input and outputs the public–private key pair $\left(pk_{i},sk_{i}\right)$ and a unique Revocation Identifier $RID_{i}$ (bound to the user for subsequent revocation operations).*

*Signature Generation: $\mathrm{Sign}(sk_{i},L_{pk},M,RID_{i})\to \sigma$, which takes a user’s private key $sk_{i}$, the ring public key set $L_{pk}=\left(pk_{1},pk_{2},\ldots ,pk_{L}\right)$, a message $M\in M_{\lambda }$, and the user’s $RID_{i}$ as inputs and outputs a linkable and revocable ring signature σ.*

*Signature Verification: $\mathrm{Ver}(L_{pk},M,\sigma ,pp)\to \mathrm{Valid}/\mathrm{Invalid}$, which takes the ring public key set $L_{pk}$, a message M, a signature σ, and the system public parameters pp as inputs and outputs Valid (the signature is legitimate and the corresponding $RID_{i}$ is not revoked) or Invalid.*

Link Detection: $\mathrm{Link}(\sigma ,\sigma^{*})\to \mathrm{Linked}/\mathrm{Unlinked}$, which takes two linkable and revocable ring signatures (corresponding to distinct messages) as inputs, compares their linking tags, and outputs Linked or Unlinked.

*Revocation Operation: $\mathrm{Revoke}(pp,RID_{i},\sigma )\to \mathrm{Revoked}/\mathrm{Not}\;\mathrm{Revoked}$, which takes the system public parameters pp, a user’s Revocation Identifier $RID_{i}$, and a suspicious signature σ as inputs and outputs Revoked (indicating the user has engaged in malicious activities) or NotRevoked.*

### 2.2. WOTS+

At present, hash-driven signature architectures, originating from Ralph Merkle’s research, are categorized into the following three classes: few-time signature (FTS), one-time signature (OTS), and many-time signature (MTS) (as shown in Table 2).

Table 1 summarizes several commonly used hash-based signature mechanisms. In one-time signature (OTS) mechanisms, the pioneering signature algorithm is the Lamport–Diffie algorithm. This study adopts the WOTS+ mechanism as the primary framework, which encompasses the following three core operational modules: Key Generation (KeyGen), Signature Generation (Sign), and Signature Verification (Ver).

The system parameters of WOTS+ include a security parameter *n* (where *n* also denotes the number of bytes for XMSS tree nodes, FORS tree nodes, and secret preimage elements), a Winternitz parameter ω, and the number of len elements in the secret preimage array (as shown in Table 3). The parameter len is calculated as $\mathrm{len}={\mathrm{len}}_{1}+{\mathrm{len}}_{2}$, where

$${\mathrm{len}}_{1}=\left\lceil \frac{8n}{\log_{2}\omega }\right\rceil ,\;{\mathrm{len}}_{2}=\left\lceil \frac{\log_{2}\left({\mathrm{len}}_{1}\cdot \left(\omega -1\right)\right)+\log_{2}\omega }{\log_{2}\omega }\right\rceil$$

**Step 1: Key Generation.**

This step derives secret preimages using a pseudorandom function (PRF) to reduce private key storage overhead, as follows: first, generate a public seed $\mathrm{PK}.\mathrm{seed}\in {\left\{0,1\right\}}^{n}$ and a private seed $\mathrm{SK}.\mathrm{seed}\in {\left\{0,1\right\}}^{n}$; then, derive the secret preimage array by computing $x_{i}=\mathrm{PRF}\left(\mathrm{SK}.\mathrm{seed},\mathrm{encode}\left(i\right)\right)$ for each i∈{0,1,…,len−1}, where encode(i) encodes *i* into a fixed length; next, construct hash chains for the public key by generating a hash chain of length ω for each $x_{i}$ with the starting point $y_{i}^{\left(0\right)}=x_{i}$ and iteratively computing the chain as $y_{i}^{\left(j+1\right)}=F\left(\mathrm{PK}.\mathrm{seed},\mathrm{ADRS}\left(i,j\right),y_{i}^{\left(j\right)}\right)$ for j∈{0,ω−2}; subsequently, assemble the public key from the final nodes of all hash chains as $\mathrm{PK}.\mathrm{pub}=\left(y_{0}^{\left(\omega -1\right)},y_{1}^{\left(\omega -1\right)},\ldots ,y_{\mathrm{len}-1}^{\left(\omega -1\right)}\right)$; and finally, output the public key pk=(PK.seed,PK.pub) and the private key sk=(PK.seed,SK.seed).

**Step 2: Signature Generation.**

This step takes the private key sk and message msg as inputs, as follows: first, convert msg into an 8n-bit string and split it into ${\mathrm{len}}_{1}$ integers $m\left[0\right],m\left[1\right],\ldots ,m\left[{\mathrm{len}}_{1}-1\right]\in \left\{0,\omega -1\right\}$ with each segment of $s=\log_{2}\omega$ bits; then, compute the checksum $\mathrm{csum}=\sum_{i=0}^{{\mathrm{len}}_{1}-1}\left(\omega -1-m\left[i\right]\right)$ and split csum into ${\mathrm{len}}_{2}$ integers $m\left[{\mathrm{len}}_{1}\right],\ldots ,m\left[\mathrm{len}-1\right]\in \left\{0,\omega -1\right\}$ with each segment of *s* bits; next, for each chain index i∈{0,1,…,len−1}, derive the chain starting point $x_{i}=\mathrm{PRF}\left(\mathrm{SK}.\mathrm{seed},\mathrm{encode}\left(i\right)\right)$, initialize the chain starting point as $y_{i}^{\left(0\right)}=x_{i}$, iteratively generate the signature node by computing $y_{i}^{\left(j+1\right)}=F\left(\mathrm{PK}.\mathrm{seed},\mathrm{ADRS}\left(i,j\right),y_{i}^{\left(j\right)}\right)$ for j∈{0,m[i]−1}, and define the signature node as ${\mathrm{sig}}_{i}=y_{i}^{m[i]}$; and finally, assemble the signature as $\mathrm{sig}=({\mathrm{sig}}_{0},{\mathrm{sig}}_{1},\ldots ,{\mathrm{sig}}_{\mathrm{len}-1})$.

**Step 3: Signature Verification.**

This step takes the public key pk=(PK.seed,PK.pub), message msg, and signature sig as inputs, as follows: first, follow the same process as the signature generation step to segment the message and calculate the checksum for obtaining the segmented array m[0..len−1]; then, for each chain index i∈{0,1,…,len−1}, extract the signature node ${\mathrm{sig}}_{i}=\mathrm{sig}\left[i\right]$, define required_iter=ω−1−m[i], initialize $z_{i}^{\left(0\right)}={\mathrm{sig}}_{i}$, iteratively compute $z_{i}^{\left(j+1\right)}=F\left(\mathrm{PK}.\mathrm{seed},\mathrm{ADRS}\left(i,m\left[i\right]+j\right),z_{i}^{\left(j\right)}\right)$ for j∈{0,required_iter−1}, and verify whether $z_{i}^{\left(\mathrm{required\_iter}\right)}=\mathrm{PK}.\mathrm{pub}\left[i\right]$ holds, where the verification succeeds if the equation is satisfied and fails otherwise.

### 2.3. FORS

This paper also adopts the FORS algorithm. FORS is a hash-based few-time signature (FTS) scheme and serves as a fundamental component of the SPHINCS+ algorithm.

First, it is necessary to define a security parameter *n* (where n∈N), which determines the length of hash values. Additionally, it is required to specify the number of FORS subtrees *k*, k∈N, the exponent α for the height of subtrees, α∈N, and the number of leaves in each subtree, which is $t=2^{\alpha }$. The parameter description of the FORS algorithm is presented in Table 4.

**Step 1: Private Key Creation.**

The private key consists of two components. The initial component is a public random seed PK.seed, which is used to distinguish different FORS instances. The second component is a secret preimage array $\left(x_{0},\ldots ,x_{kt-1}\right)$, containing k×t *n*-byte random numbers divided into *k* groups with *t* elements each.

**Step 2: Public Key Generation.**

This step includes the following three sub-steps: computing leaf nodes, constructing FORS subtrees, and compressing root nodes into the public key. Compute leaf nodes: For each secret preimage $x_{j}$, j∈{0,k×t−1}, generate a leaf node $y_{j}$, $y_{j}=F\left(\mathrm{PK}.\mathrm{seed},\mathrm{ADRS}\left(j\right),x_{j}\right)$. Here, ADRS(j) uniquely identifies the address parameter of the *j*-th leaf. Construct FORS subtrees: Divide the k×t leaves into *k* groups with *t* leaves each, and construct a binary tree of height α. If node *z* is the parent of child nodes *u* and *v* (with the parent node having height treeHeight and index treeIndex), then z=H(PK.seed,ADRS(treeHeight,treeIndex),u ‖ v). Compress root nodes into the public key: Let the roots of the *k* subtrees be ${\mathrm{root}}_{0}^{\mathrm{FORS}},{\mathrm{root}}_{1}^{\mathrm{FORS}},\ldots ,{\mathrm{root}}_{k-1}^{\mathrm{FORS}}$. Compute the public key using the hash function $T_{k}$: $\mathrm{pk}=\left(\mathrm{PK}.\mathrm{seed},T_{k}\left(\mathrm{PK}.\mathrm{seed},{\mathrm{root}}_{0}^{\mathrm{FORS}}\left\|\;\cdots \;\right\|{\mathrm{root}}_{k-1}^{\mathrm{FORS}}\right)\right)$.

**Step 3: Signature Generation.**

This step includes the following two sub-steps: message preprocessing combined with the SPHINCS+ framework and extracting signature content. Message preprocessing: First, generate a random number $R={\mathrm{PRF}}_{\mathrm{msg}}\left(\mathrm{SK}.\mathrm{prf},\mathrm{opt},\mathrm{msg}\right)$, where ${\mathrm{PRF}}_{\mathrm{msg}}$ is a pseudorandom function (PRF) and SK.prf is a secret seed. Next, compute the message digest $=H_{\mathrm{msg}}\left(R,\mathrm{PK}.\mathrm{seed},\mathrm{PK}.\mathrm{Root},\mathrm{msg}\right)$, where *H* is a hash function. Finally, extract indices: Take the first k×α bits of the digest and split them into *k* integers $m_{0},m_{1},\ldots ,m_{k-1}$, corresponding to the leaf index of the *i*-th subtree. Extract signature content: The signature contains two types of data. The first type includes *k* secret preimages $x_{i\times t+m_{i}}$, each corresponding to the leaf with index $m_{i}$ in the *i*-th subtree. The second type is the intermediate nodes (i.e., authentication paths) traversed from the leaf $y_{i\times t+m_{i}}$ to the root ${\mathrm{root}}_{i}^{\mathrm{FORS}}$ in each subtree.

**Step 4: Signature Verification.**

**Figure 2:**
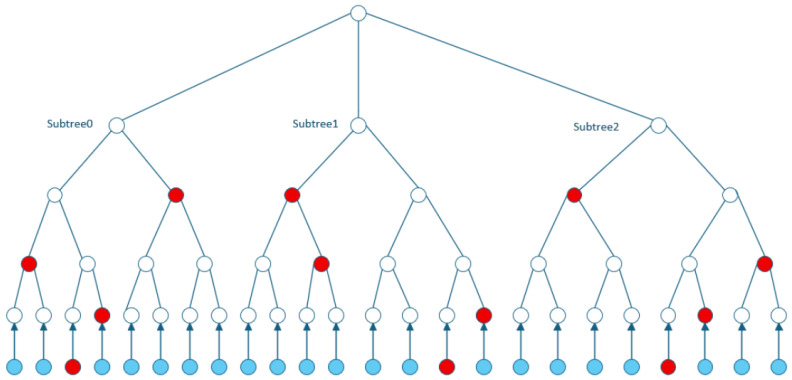
FORS instance with parameters $\left(n,k,t=2^{3}\right)$.

This step includes the following two sub-steps: reconstructing subtree root nodes and verifying public key consistency. Reconstruct subtree root nodes: For each secret preimage $x_{i\times t+m_{i}}$ and authentication path in the signature, compute the leaf $y_{i\times t+m_{i}}^{\prime }=F\left(\mathrm{PK}.\mathrm{seed},\mathrm{ADRS}\left(i\times t+m_{i}\right),x_{i\times t+m_{i}}\right)$ to verify leaf consistency. Then, simulate the subtree construction process using the intermediate nodes of the authentication path to compute the reconstructed root ${\mathrm{root}}_{i}^{{\mathrm{FORS}}^{\prime }}$. Verify the consistency of the public key: First, compress the reconstructed roots. Use all reconstructed roots ${\mathrm{root}}_{0}^{{\mathrm{FORS}}^{\prime }},\ldots ,{\mathrm{root}}_{k-1}^{{\mathrm{FORS}}^{\prime }}$ to recompute the public key via $T_{k}$: ${\mathrm{pk}}^{\prime }=\left(\mathrm{PK}.\mathrm{seed},T_{k}\left(\mathrm{PK}.\mathrm{seed},{\mathrm{root}}_{0}^{{\mathrm{FORS}}^{\prime }}\left\|\;\cdots \;\right\|{\mathrm{root}}_{k-1}^{{\mathrm{FORS}}^{\prime }}\right)\right)$. The signature is valid if ${\mathrm{pk}}^{\prime }$ is exactly consistent with the original public key. Figure 2 (blue nodes at the bottom layer are the secret key leaves serving as private key sources for signing, red nodes are the authentication path nodes corresponding to the selected private key that are provided in the signature for root reconstruction during verification, and white nodes are the non-authentication path intermediate nodes only used to construct the tree structure without being included in the signature.) presents the tree structure of a FORS instance with parameters $\left(n,k,t=2^{3}\right)$, as well as the private key elements and authentication path required when signing the message M=010110100=(2,6,4).

### 2.4. SPHINCS+

#### 2.4.1. XMSS

**Figure 3:**
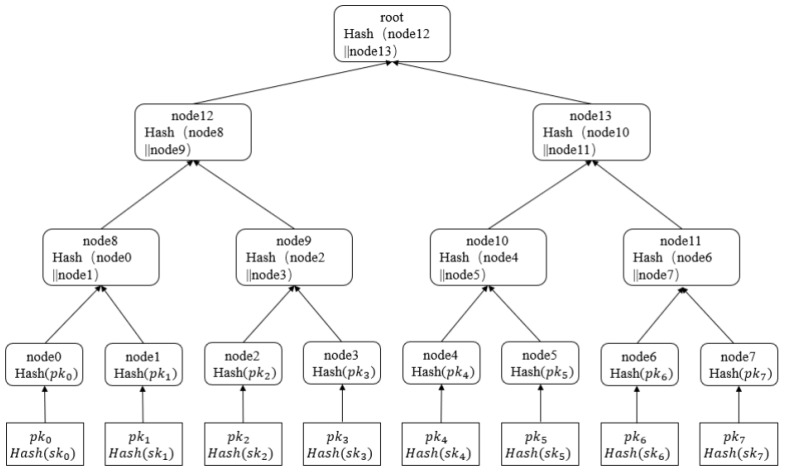
Structure of the Merkle tree.

In 1979, Ralph Merkle put forward the Merkle Signature Scheme (MSS), which integrates Merkle trees with one-time signature (OTS) algorithms. The Merkle structure exhibits a layered architecture in which leaf elements store hash digests of information, whereas non-leaf elements maintain aggregated hash digests of their subordinate elements. Such an architecture facilitates effective validation of information authenticity and also proves particularly apt for extensive data collections. The overall architecture of the Merkle structure is illustrated in Figure 3.

Depicted in Figure 3, the structure comprises three tiers and $2^{3}=8$ leaf elements, each storing the hash digest of a single-use signature public key. The leaf elements are designated from elem0 to elem7 and are hashed in pairs to generate intermediate elements. The final root element stores the public key. Merkle structures mainly fulfill the following two functions:

Information authenticity validation: Individuals can ascertain if the information has been modified by recomputing the root digest.

Public key dimension reduction: By combining numerous public keys into a single root key, it reduces the storage requirement for public keys.

**Figure 4:**
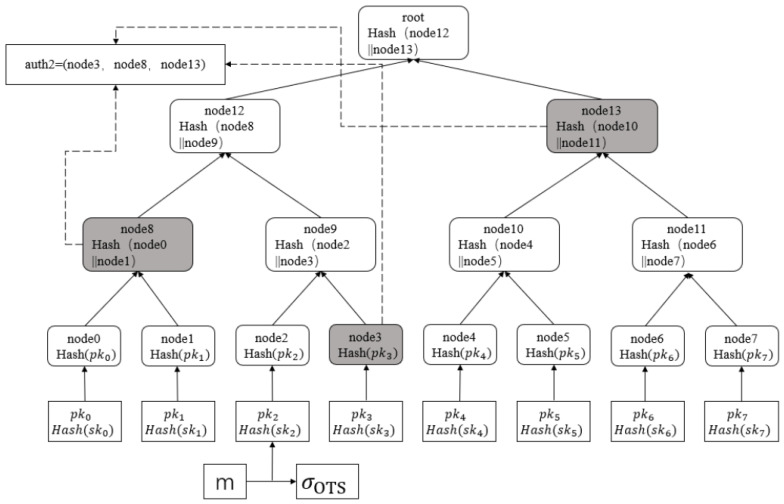
Signature generation process.

XMSS (eXtended Merkle Signature Scheme) is an extended Merkle signature scheme. It is a hash-based digital signature system with WOTS+ as its primary building block, managing WOTS+ keys through multi-layered Merkle trees. An *h*-height Merkle tree contains $2^{h}$ WOTS+ public keys, with its root node serving as the XMSS public key. During signing, an unused leaf index is selected, the corresponding WOTS+ key is used to generate the signature, and path nodes are appended (as shown in Figure 4, gray boxes represent core nodes on the authentication path, while white boxes represent non-authentication path nodes and basic components of the XMSS tree). During verification, the hash chain of the auth2 nodes in the path is computed, the root is reconstructed, and is compared with the public key.

Being a quantum-resistant hash-centric digital signing mechanism, SPHINCS+ adopts relevant XMSS technologies during its construction and improves upon some of XMSS’s drawbacks. XMSS possesses a stateful property, which means that to ensure overall security, it needs to track all signatures generated with the same private key. This property limits the attractiveness of its practical applications to a certain extent. In contrast, SPHINCS+ overcomes the limitations caused by XMSS’s statefulness through improvements and adopts a stateless design.

#### 2.4.2. Hypertree

**Figure 5:**
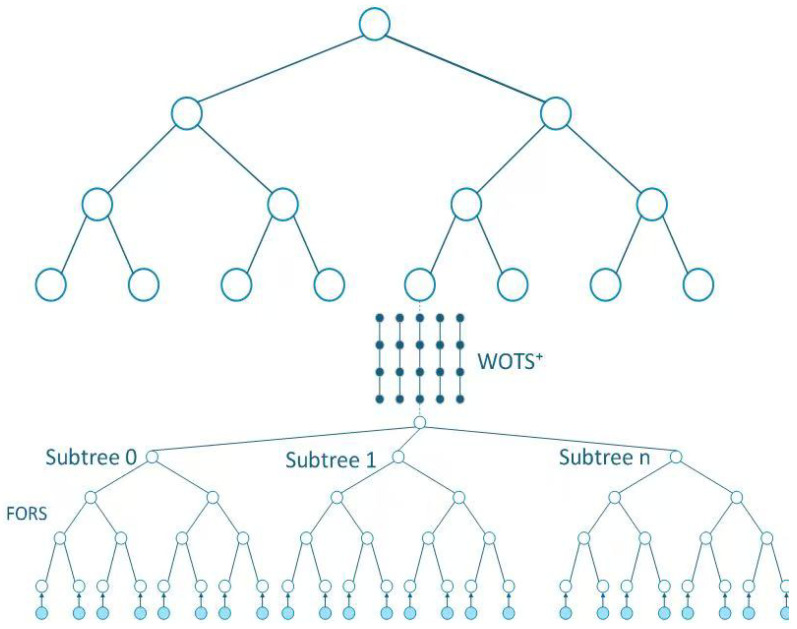
Structure of the Hypertree.

As the core framework for organizing WOTS+ one-time signature and FORS few-time signature components, the Hypertree of SPHINCS+ consists of multiple layers of XMSS trees. The root value of each layer’s tree is used to verify the public keys of the next layer, eliminating key state management issues. The structure of the Hypertree is illustrated in Figure 5.

The Hypertree is composed of a large number of perfect binary trees (variants of XMSS trees) with the same height, arranged in a hierarchical manner. It has a total height *h* and is divided into *d* layers, where the height of each XMSS tree is $h^{\prime }=\frac{h}{d}$. Except for the topmost layer, any XMSS tree in the *i*-th layer corresponds to a leaf node of a specific XMSS tree in the (i+1)-th layer, forming a hierarchical association.

The topmost layer (the (d−1)-th layer) contains only $2^{0}=1$ XMSS tree. From the upper to lower layers, the number of XMSS trees increases layer by layer, with the bottommost layer (the 0-th layer) having $2^{\left(h-h^{\prime }\right)}$ XMSS trees. Each XMSS tree contains $2^{h^{\prime }}$ leaf nodes, and the datum of every leaf element represents the condensed datum of the public key set for a WOTS+ entity. The leaf elements of all XMSS structures at the lowermost layer correspond to a total of $2^{h}$ WOTS+ instances and $2^{h}$ FORS instances.

The index of a specific leaf node in the bottommost layer (the 0-th layer) can activate a set of “XMSS tree chains” and “XMSS leaf node chains”. A tree chain includes XMSS trees from the 0-th layer to the (d−1)-th layer, where the tree in the *i*-th layer corresponds to a specific leaf node of the tree in the (i+1)-th layer. A leaf node chain includes the corresponding leaf nodes of the XMSS trees in each layer of the aforementioned tree chain, serving as the hierarchical verification path during signature generation.

The Hypertree structure supports signature generation through tree chains and leaf node chains. During signature generation, the index of the XMSS tree and leaf node in the bottommost layer is first determined via the message digest, activating the corresponding tree chain and leaf node chain. Then, XMSS signatures are generated layer by layer from the bottommost layer to the topmost layer, eventually forming the Hypertree signature.

## 3. SPHINCS+ Linkable and Revocable Ring Signature Scheme

The interaction process among V2G entities (EV, CS, LAG, BS, Smart Grid Control Center (SGCC), TA) involves multiple wireless/wired communication links and hierarchical data processing, leading to scenario-specific security risks that require focused attention in security analysis, as follows: (1) the wireless communication between EVs and LAGs is vulnerable to man-in-the-middle attacks, where attackers may tamper with charging requests (e.g., modifying electricity demand *q* or transaction amount *v*) or hijack signature data; (2) as an intermediate node for signature verification and transaction forwarding, LAGs may be compromised to forge verification results or delay transaction synchronization, creating opportunities for malicious EVs to conduct double-spending; (3) CSs may falsify charging records to collude with users in fraudulent transactions, requiring the scheme to bind transaction data with immutable signatures; (4) the linkable tags I stored by BSs may become targets for tampering, undermining the double-spending detection mechanism; and (5) the on-board terminals of EVs are resource-constrained and susceptible to side-channel attacks such as power analysis and timing analysis, necessitating enhanced attack resistance in the scheme design. Subsequent security analysis will design attack scenarios targeting these links to ensure the scheme covers end-to-end risks.

### 3.1. Description of the Scheme’s Signature Algorithm

The hash-centric linkable ring signing mechanism addressed in this study relies on the SPHINCS+ mechanism, comprising the following three modules: the WOTS+ one-time signature algorithm, the FORS few-time signature algorithm, and the XMSS hierarchical tree algorithm. The following is an overview of these components.

**Definition** **2** **(SPHINCS+-based Linkable Ring Signature Algorithm).** *The SPHINCS+-based linkable ring signature algorithm mainly includes the following five steps: Initialization, Key Generation, Signature Generation, Signature Verification, and Link Detection. The algorithms for these steps are as follows:*

**Step 1: Initialization.**

Input the security parameter λ and output the public parameters $\mathrm{pp}=(H,H_{\mathrm{tree}},\omega ,\mathrm{len},k,h,d,n_{\mathrm{ring},\max},{\mathrm{Root}}_{\mathrm{ring}},{\mathrm{auth}}_{\mathrm{ring}},\tau_{\mathrm{win}})$. Determine the hash functions $H:{\left\{0,1\right\}}^{*}\to {\left\{0,1\right\}}^{n}$ and $H_{\mathrm{tree}}:{\left\{0,1\right\}}^{*}\to {\left\{0,1\right\}}^{n}$, as well as the maximum ring size $n_{\mathrm{ring},\max}$. In real-world implementation contexts, if the quantity of vehicles fails to satisfy this criterion, it is advisable to either incorporate virtual entities into the ring or segment the vehicles into several rings. The parameter $\tau_{\mathrm{win}}$ (time window interval, e.g., 3600 s) maps transaction timestamp to discrete time window $t=\lfloor \mathrm{timestamp}/\tau_{\mathrm{win}}\rfloor$ ($t\in N^{+}$) for forward security implementation.

See Table 5 for detailed parameter descriptions.

**Step 2: Key Generation.**

Input the public parameters pp and output the user private key $sk_{i}$, public key $pk_{i}$, and linkage auxiliary parameters. The specific process is as follows: First, generate the SPHINCS+ private key $sk_{i}=\left({\mathrm{SK}.\mathrm{seed}}_{i},{\mathrm{SK}.\mathrm{prf}}_{i}\right)$ and public key $pk_{i}=\left({\mathrm{PK}.\mathrm{root}}_{i},{\mathrm{PK}.\mathrm{seed}}_{i}\right)$; subsequently, generate the initial linkage private key $sk_{\mathrm{link},i,0}=H({\mathrm{SK}.\mathrm{seed}}_{i}\|\;“link\_init”\;\|\tau_{0})$, as well as the public key hash $hpk_{i}=H\left(pk_{i}\right)$.

**Step 3: Signature Generation.**

Input the message *M*, amount *v*, ring member set $R=\{PK_{1},...,PK_{n_{\mathrm{ring}}}\}$, and the signer’s private key $sk_{\xi }$ (where $\xi \in \{1,\ldots ,n_{\mathrm{ring}}\}$), and output the linkable ring signature σ.

The signer first determines the current time window $t=\lfloor \mathrm{timestamp}/\tau_{\mathrm{win}}\rfloor$ from the transaction timestamp, then updates the linkage private key in a one-way manner for forward security: $sk_{\mathrm{link},\xi ,t}=H(sk_{\mathrm{link},\xi ,t-1}\|\;t\;\|\;H\left(PK_{\xi }\;\|\;\tau_{t}\right))$.

The signer maps the ring member public key set $R=\{PK_{1},...,PK_{n_{\mathrm{ring}}}\}$ to a hash set $\left\{H\left(PK_{1}\right),...,H\left(PK_{n_{\mathrm{ring}}}\right)\right\}$, constructs a Merkle tree using this set as leaf nodes, and calculates the root node ${\mathrm{Root}}_{\mathrm{ring}}=H_{\mathrm{tree}}\left(\{H\left(PK_{1}\right),\ldots ,H\left(PK_{n_{\mathrm{ring}}}\right)\}\right)$.

Extract the authentication path ${\mathrm{auth}}_{\mathrm{ring}}$ from the hash of the signer’s own public key $H(PK_{\xi })$ to ${\mathrm{Root}}_{\mathrm{ring}}$; the path length is $\log_{2}n_{\mathrm{ring}}$, covering all intermediate nodes from the leaf (i.e., $H(PK_{\xi })$) to the root ${\mathrm{Root}}_{\mathrm{ring}}$.

Randomly select $r\in {\left\{0,1\right\}}^{n}$ and compute $\mathrm{cm}=H\left(r\;\|\;v\;\|\;H\left(PK_{\xi }\right)\;\|\;H\left(M\right)\right)$, which binds the transaction amount and the signer.

Randomly select an independent random factor $r_{\xi ,t}\in {\left\{0,1\right\}}^{n}$, then compute the forward-secure link tag for subsequent link detection: $I_{\xi ,t}=H\left(sk_{\mathrm{link},\xi ,t}\;\|\;H\left(PK_{\xi }\right)\;\|\;t\;\|\;r_{\xi ,t}\right)$ Perform message preprocessing: Let $M^{\prime }=c\;\|\;I_{\xi ,t}$, and generate the digest $md=H(M^{\prime })$. For each non-signer i≠ξ in the ring, generate indistinguishable pseudo-FORS signatures. Pseudo-FORS private key generation: $sk_{\mathrm{For},i}^{\mathrm{fk}}=\mathrm{PRF}\left({\mathrm{PK}.\mathrm{sd}}_{r},\mathrm{enc}(i\;\|\;t\;\|\;{\mathrm{Adr}}_{\mathrm{For},i})\right)$; Pseudo-FORS authentication path generation: ${\mathrm{auth}}_{\mathrm{For},i}^{\mathrm{fk}}=\left\{H(\mathrm{PK}.\mathrm{sd},{\mathrm{Adr}}_{n,j},r_{j})|j=1,\ldots ,\alpha \right\}$; Pseudo-FORS root consistency verification: ${\mathrm{root}}_{\mathrm{For},i}^{\mathrm{fk}}=T_{k}\left({\mathrm{PK}.\mathrm{sd}}_{i},{\mathrm{Adr}}_{\mathrm{rt}},{\mathrm{auth}}_{\mathrm{For},i}^{\mathrm{fk}}\right)$; Pseudo-FORS signature construction: $\sigma_{\mathrm{For},i}^{\mathrm{fk}}=\left(sk_{\mathrm{For},i}^{\mathrm{fk}}\left[m\left[i\right]\right],{\mathrm{auth}}_{\mathrm{For},i}^{\mathrm{fk}},{\mathrm{Adr}}_{\mathrm{For},i}\right)$.

Use the real FORS private key $sk_{\mathrm{FORS},\xi [j]}=\mathrm{PRF}\left({\mathrm{PK}.\mathrm{seed}}_{\xi },{\mathrm{SK}.\mathrm{seed}}_{\xi },\mathrm{ADRS}\right)$ (where j∈{1,...,k−t}) to generate a valid signature $\sigma_{\mathrm{FORS},\xi }$ for md. Aggregate the FORS signatures: $\sigma_{\mathrm{FORS}}=\left\{\sigma_{\mathrm{FORS},1},\ldots ,\sigma_{\mathrm{FORS},n_{\mathrm{ring}}}\right\}$.

Generate the WOTS+ private key $sk_{\mathrm{WOTS}+,\xi [i]}=\mathrm{PRF}\left({\mathrm{PK}.\mathrm{seed}}_{\xi },{\mathrm{SK}.\mathrm{seed}}_{\xi },\mathrm{ADRS}\right)$ (where i∈{0,...,len−1}). Take $\sigma_{\mathrm{FORS}}$ as the input of the WOTS+ algorithm, and use $sk_{\mathrm{WOTS}+,\xi [i]}$ to sign $\sigma_{\mathrm{FORS}}$, generating $\sigma_{\mathrm{WOTS}+}$.

Locate the leaf node of ξ in the XMSS tree (with index idxLeaf) and extract the authentication path ${\mathrm{auth}}_{\mathrm{XMSS}}$ from this leaf node to the tree root ${\mathrm{PK}.\mathrm{root}}_{\xi }$.

Incorporate ${\mathrm{Root}}_{\mathrm{ring}}$ and ${\mathrm{auth}}_{\mathrm{ring}}$ into the signature structure.

Finally, output $\sigma =\left(\sigma_{\mathrm{FORS}},\sigma_{\mathrm{WOTS}+},{\mathrm{auth}}_{\mathrm{XMSS}},{\mathrm{auth}}_{\mathrm{ring}},\mathrm{cm},I_{\xi ,t},r,r_{\xi ,t},t,{\mathrm{Root}}_{\mathrm{ring}}\right)$.

**Step 4: Signature Verification.**

Input the signature σ, message *M*, ring *R*, and public parameters pp, and output the verification result 0 (invalid) or 1 (valid).

Extract ${\mathrm{Root}}_{\mathrm{ring}}$, ${\mathrm{auth}}_{\mathrm{ring}}$, and the hash of the signer’s public key $H(PK_{\xi })$ from the signature σ.

Starting from $H(PK_{\xi })$, iteratively hash the intermediate nodes contained in ${\mathrm{auth}}_{\mathrm{ring}}$ to reconstruct the Merkle root ${\mathrm{Root}}_{\mathrm{ring}}^{\prime }$.

If ${\mathrm{Root}}_{\mathrm{ring}}^{\prime }\neq {\mathrm{Root}}_{\mathrm{ring}}$, return 0; if they are consistent, proceed to the subsequent verification steps.

Verify the hash commitment: Extract cm and *r*, and verify $H\left(r\;\|\;v\;\|\;H\left(PK_{\xi }\right)\;\|\;H\left(M\right)\right)=\mathrm{cm}$. If this verification fails, return 0.

Verify the FORS signature: For each $\sigma_{\mathrm{FORS},i}$ in $\sigma_{\mathrm{FORS}}$, use ${\mathrm{PK}.\mathrm{seed}}_{i}$ and the authentication paths in $\sigma_{\mathrm{FORS},i}$ to verify the FORS tree root ${\mathrm{root}}_{\mathrm{FORS}}=T_{k}\left({\mathrm{PK}.\mathrm{seed}}_{i},\mathrm{ADRS},{\mathrm{root}}_{j}\right)$; confirm it is valid if the verification passes for $pk_{i}$.

Verify the WOTS+ signature: For $pk_{i}$ that passes the FORS verification, use ${\mathrm{PK}.\mathrm{seed}}_{i}$ and $\sigma_{\mathrm{WOTS}+}$ to verify the WOTS+ public key $pk_{\mathrm{WOTS}+}=T_{\mathrm{len}}\left({\mathrm{PK}.\mathrm{seed}}_{i},\mathrm{ADRS},y_{i}^{\omega -1}\right)$, where $y_{i}^{\omega -1}$ are the final nodes of the hash chains of the *i*-th group of WOTS+ private keys. If the verification passes, $\sigma_{\mathrm{WOTS}+}$ is not tampered with.

Verify the XMSS path: Use ${\mathrm{auth}}_{\mathrm{XMSS}}$ and ${\mathrm{PK}.\mathrm{root}}_{i}$ to verify the path integrity, compute layer by layer up to the XMSS root, and confirm consistency with ${\mathrm{PK}.\mathrm{root}}_{i}$.

Extract $I_{\xi ,t}$, $r_{\xi ,t}$, *t*, and $sk_{\mathrm{link},\xi ,t}$ and verify the forward-secure link tag consistency: $I_{\xi ,t}=H\left(sk_{\mathrm{link},\xi ,t}\;\|\;H\left(PK_{\xi }\right)\;\|\;t\;\|\;r_{\xi ,t}\right)$. If verification fails, return 0.

Return 1 if all steps pass; otherwise, return 0.

**Step 5: Link Detection.**

Input two signatures $\sigma_{1},\sigma_{2}$, and output “linked” or “unlinked”.

Extract $I_{\xi_{1},t_{1}}$, $I_{\xi_{2},t_{2}}$ from $\sigma_{1}$, $\sigma_{2}$, along with $r_{\xi_{1},t_{1}}$, $r_{\xi_{2},t_{2}}$, $t_{1}$, $t_{2}$ and the passed verification results.

Return “linked” if $I_{\xi_{1},t_{1}}=I_{\xi_{2},t_{2}}$ and $t_{1}=t_{2}$; otherwise, return “unlinked”.

### 3.2. Application of the Scheme in Vehicle-to-Grid (V2G) Networks

This paper applies the SPHINCS+-based linkable ring signature algorithm to the anonymous payment scenario in Vehicle-to-Grid (V2G) networks. Based on the post-quantum signature mechanism of SPHINCS+ (core parameters: k=16, α=8, ω=16, $\tau_{\mathrm{win}}=3600\;s$), the scheme integrates the anonymity and linkability of ring signatures and innovatively designs a **KEK-based collaborative revocation mechanism** to balance privacy protection and regulatory efficiency. It protects the identity privacy of Electric Vehicles (EVs), supports the associative traceability of multiple transactions from the same user, effectively resists double-spending attacks, and enables secure revocation of malicious nodes without privacy leakage.

The scheme involves the following seven core entities: Trusted Authority (TA), Electric Vehicle (EV), Charging Station (CS), Local Aggregator (LAG), Smart Grid Control Center (SGCC), Billing Server (BS), and Independent Regulatory Node (RN). The TA is responsible for system initialization, key management, and collaborative revocation; the RN holds a fragment of the Key Encryption Key (KEK) to constrain the TA’s authority; and other entities perform transaction initiation, verification, and execution as specified. Each EV must complete registration with the TA to obtain a unique Revocation Identifier (RID) and access the network.

When an EV needs to purchase electricity for charging, it initiates a charging request containing its anonymous identifier to the LAG; the LAG forwards the request to the SGCC for confirmation; the SGCC generates an order and returns it to the EV; the EV selects n−1 registered EVs to form a ring, generates a SPHINCS+-based linkable ring signature, and sends it to the BS via the LAG; and the BS verifies the signature validity and authorizes charging if passed.

This section elaborates on the anonymous payment mechanism in detail, including four core steps (initialization, account preparation, signature payment, verification and authorization) and a supporting traceability–revocation process. Assume an EV user (Alice) has *m* accounts and intends to pay for V2G charging through this mechanism.

**Figure 6:**
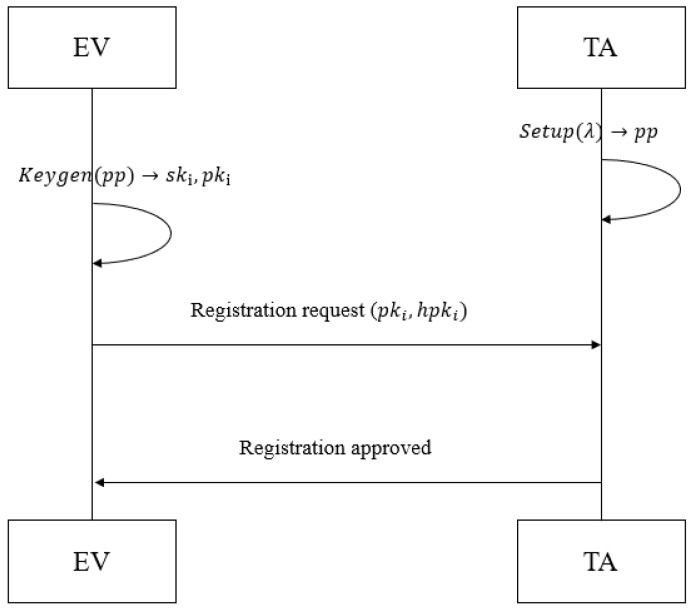
Initialization process (including KEK sharding).

**Step 1: Initialization (See Figure 6).**

First, the TA executes the SPHINCS+ Setup(λ) algorithm to generate system parameters $pp=(H,H_{\mathrm{tree}},\omega ,\mathrm{len},k,\alpha ,h,d,n_{\mathrm{ring},\max},{\mathrm{PK}.\mathrm{seed}}_{\mathrm{rand}},\tau_{\mathrm{win}})$ (where *H* is SHA-256 and $H_{\mathrm{tree}}$ is a Merkle tree hash function) and distributes pp as the global public parameter to all entities. Subsequently, the TA generates a Key Encryption Key (KEK) using the AES-256 algorithm and splits it into the following two fragments via XOR operation: $KEK=KEK_{1}⊕KEK_{2}$, where $KEK_{1}$ is stored locally by the TA and $KEK_{2}$ is held by the independent Regulatory Node (RN) (realizing collaborative decryption constraint). Alice submits an encrypted registration application to the TA (containing device identifier and identity verification information), executes KeyGen(pp) to generate a public–private key pair $\left(pk_{i},sk_{i}\right)$, and computes the following. Initial linkage private key: $sk_{\mathrm{link},i,0}=H({\mathrm{SK}.\mathrm{seed}}_{i}\|\;“link_{i}nit”\;\|\;\tau_{0})$ ($\tau_{0}$ is registration timestamp, ${\mathrm{SK}.\mathrm{seed}}_{i}$ is SPHINCS+ private seed). Public key hash: $hpk_{i}=H\left(pk_{i}\right)$ (ensuring consistency with signature verification logic). The TA assigns a unique unforgeable Revocation Identifier $RID_{i}$ to Alice, constructs a mapping table entry $\left(I_{\mathrm{link}},pk_{i},RID_{i}\right)$ (where $I_{\mathrm{link}}$ is the initial link tag), encrypts the entire mapping table with KEK (AES-256), and stores it in regional fragments (impact scope of single leakage $\leq \frac{1}{n_{\mathrm{shard}}}$, $n_{\mathrm{shard}}$ is fragment count). The TA generates a revocation credential $Cred_{RID_{i}}=H(RID_{i}\;\|\;\mathrm{PK}.\mathrm{root}\;\|\;\tau_{0})$ (binding RID with SPHINCS+ public root), adds $pk_{i}$ and $RID_{i}$ to the legitimate user list, and Alice stores $RID_{i}$, $Cred_{RID_{i}}$, and $sk_{i}$ locally.

**Figure 7:**
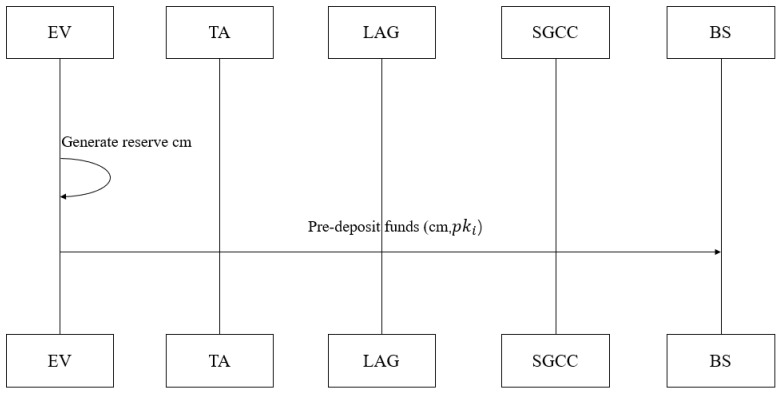
Account creation process.

**Step 2: Account Creation Process (See Figure 7).**

Before participating in transactions, Alice needs to create an account and pre-deposit funds to ensure transaction credibility. First, she generates a random number $r\in {\left\{0,1\right\}}^{256}$ as the commitment factor and computes the balance commitment $cm=H(r\;\|\;v\;\|\;hpk_{i}\;\|\;H\left(M\right)\;\|\;RID_{i})$—where *v* is the account balance, *M* is the initial transaction message, and $RID_{i}$ binds the account with the revocation mechanism (consistent with SPHINCS+ hash input length). Account information is stored in the form of $\left(pk_{i},cm,RID_{i}\right)$: the hash commitment hides the actual balance while ensuring verifiability, and the association between the account and revocation mechanism is realized through $RID_{i}$; the TA synchronizes the encrypted account mapping table fragment to the BS, ensuring only authorized entities can access it.

**Figure 8:**
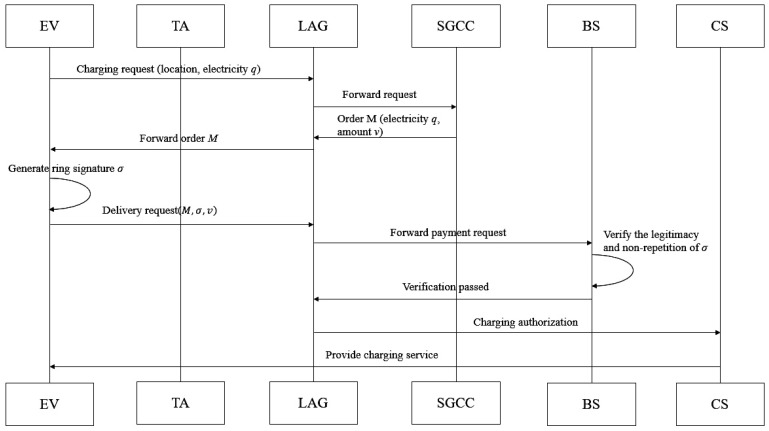
Anonymous payment phase.

**Step 3: Anonymous Payment Phase (See Figure 8).**

First, Alice submits a charging request to the LAG, including the CS location, electricity demand *q*, anonymous identifier, and revocation credential $Cred_{RID_{i}}$. The LAG forwards the request to the SGCC, which computes the electricity fee *v*, constructs the bill message M=(EVAnonymousID,ElectricityQuantity,v,Timestamp), and returns it to Alice.

Next, Alice selects n−1 legitimate EV public key hashes $hpk_{j}$ (from the TA’s published list) and forms a ring $R=\{hpk_{1},...,hpk_{\xi },...,hpk_{n}\}$ with her own $hpk_{\xi }$ (ξ is her index in the ring), ensuring the ring size $n\leq n_{\mathrm{ring},\max}$.

When generating the signature, Alice strictly follows the scheme’s parameter constraints and pseudo-signature generation logic. Time window determination: Compute the current time window $t=\lfloor \mathrm{Timestamp}/\tau_{\mathrm{win}}\rfloor$ and update the linkage private key in one-way manner (ensuring forward secrecy), $sk_{\mathrm{link},\xi ,t}=H(sk_{\mathrm{link},\xi ,t-1}\;\|\;t\;\|\;H\left(pk_{\xi }\;\|\;\tau_{t}\right))$ ($\tau_{t}$ is the start timestamp of time window *t*). Ring Merkle tree construction: Take ring *R* as leaf nodes and compute Merkle root ${\mathrm{Root}}_{\mathrm{ring}}=H_{\mathrm{tree}}\left(R\right)$ and authentication path ${\mathrm{auth}}_{\mathrm{ring}}$ (associating $hpk_{\xi }$ with ${\mathrm{Root}}_{\mathrm{ring}}$), ensuring ring member legitimacy verification. Commitment and link tag generation: Generate commitment $cm=H(r\;\|\;v\;\|\;hpk_{\xi }\;\|\;H\left(M\right)\;\|\;RID_{i})$, select a one-time random factor $tk_{\xi ,t}\in {\left\{0,1\right\}}^{256}$ (tk = transaction key), and compute forward-secure link tag $I_{\xi ,t}=H(sk_{\mathrm{link},\xi ,t}\;\|\;hpk_{\xi }\;\|\;t\;\|\;tk_{\xi ,t}\;\|\;RID_{i})$. Pseudo-signature generation for non-signers (ensuring polynomial indistinguishability): For each i≠ξ in the ring: The pseudo-FORS private key is $sk_{\mathrm{FORS},i}^{\mathrm{fake}}=\mathrm{PRF}({\mathrm{PK}.\mathrm{seed}}_{\mathrm{rand}},\mathrm{encode}(i\;\|\;t\;\|\;{\mathrm{ADRS}}_{\mathrm{FORS},i}))$ (consistent with SPHINCS+ address encoding) and pseudo-authentication path is ${\mathrm{auth}}_{\mathrm{FORS},i}^{\mathrm{fake}}=\left\{H\left(\mathrm{PK}.\mathrm{seed},{\mathrm{ADRS}}_{\mathrm{node},j},rand_{j}\right)|j=1,\ldots ,\alpha \right\}$ (authentication path length α=8). Root consistency verification: ${\mathrm{root}}_{\mathrm{FORS},i}^{\mathrm{fake}}=T_{k}\left({\mathrm{PK}.\mathrm{seed}}_{i},{\mathrm{ADRS}}_{\mathrm{root}},{\mathrm{auth}}_{\mathrm{FORS},i}^{\mathrm{fake}}\right)$ (FORS subtrees k=16). Pseudo-signature construction: $\sigma_{\mathrm{FORS},i}^{\mathrm{fake}}=\left(sk_{\mathrm{FORS},i}^{\mathrm{fake}}\left[m\left[i\right]\right],{\mathrm{auth}}_{\mathrm{FORS},i}^{\mathrm{fake}},{\mathrm{ADRS}}_{\mathrm{FORS},i}\right)$ (hash distribution calibrated to $|\mu_{\mathrm{fake}}-\mu_{\mathrm{real}}|\leq 0.01$). Real signature generation for signer: Use real FORS private key $sk_{\mathrm{FORS},\xi [j]}=\mathrm{PRF}\left({\mathrm{PK}.\mathrm{seed}}_{\xi },{\mathrm{SK}.\mathrm{seed}}_{\xi },\mathrm{ADRS}\right)$ (j∈{1,...,k·t}, $t=2^{\alpha }=256$) to generate a valid signature $\sigma_{\mathrm{FORS},\xi }$ for digest $md=H(cm\;\|\;I_{\xi ,t})$, generate a WOTS+ signature $\sigma_{\mathrm{WOTS}+}$ (len=67 segments) to bind $\sigma_{\mathrm{FORS}}$, and extract the XMSS authentication path ${\mathrm{auth}}_{\mathrm{XMSS}}$ (subtree height $h^{\prime }=10$). Signature aggregation: Output the complete signature $\sigma =(\sigma_{\mathrm{FORS}},\sigma_{\mathrm{WOTS}+},{\mathrm{auth}}_{\mathrm{XMSS}},{\mathrm{auth}}_{\mathrm{ring}},cm,I_{\xi ,t},r,tk_{\xi ,t},t,{\mathrm{Root}}_{\mathrm{ring}},Cred_{RID_{i}})$ and send *M*, σ, *v*, cm to the LAG, which forwards to the BS.

In the verification and authorization phase, the BS executes multi-layer verification with consistent parameters. Ring legitimacy verification: Reconstruct Merkle root ${\mathrm{Root}}_{\mathrm{ring}}^{\prime }$ via $hpk_{\xi }$ and ${\mathrm{auth}}_{\mathrm{ring}}$ and check consistency with ${\mathrm{Root}}_{\mathrm{ring}}$. Credential validity verification: Verify $Cred_{RID_{i}}=H(RID_{i}\;\|\;\mathrm{PK}.\mathrm{root}\;\|\;\tau_{0})$ via the TA’s legitimate list, excluding revoked nodes. Signature component verification: Verify FORS/WOTS+/XMSS components (consistent with SPHINCS+ verification logic), ensuring no tampering. Link tag consistency verification: Check $I_{\xi ,t}=H(sk_{\mathrm{link},\xi ,t}\;\|\;hpk_{\xi }\;\|\;t\;\|\;tk_{\xi ,t}\;\|\;RID_{i})$, ensuring traceability. Authorization execution: If all verifications pass, notify LAG to authorize CS charging and record $I_{\xi ,t}$, *t*, $RID_{i}$ for double-spending detection.

**Figure 9:**
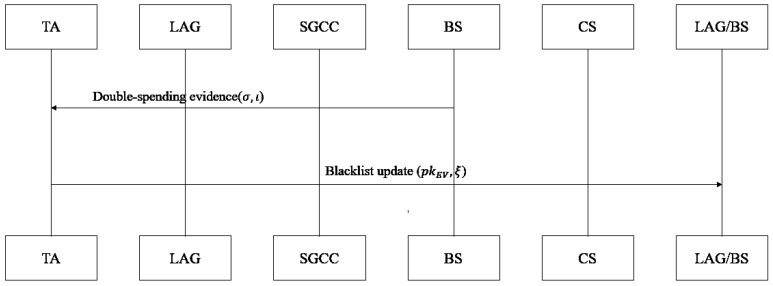
Traceability and revocation phase.

**Step 4: Traceability and Revocation Phase (See Figure 9).**

The BS monitors transaction records in real time: if a duplicate $I_{\xi ,t}$ is detected in the same *t* (double-spending) or malicious behavior is identified, it reports σ, $I_{\xi ,t}$, *t*, $RID_{i}$ to the TA; the TA initiates collaborative decryption, requesting $KEK_{2}$ from the RN, reconstructing complete $KEK=KEK_{1}⊕KEK_{2}$, decrypting the regional fragment of the $\left(I_{\mathrm{link}},pk,RID\right)$ mapping table (AES-256 decryption), and obtaining the association between $RID_{i}$ and $pk_{EV,\xi }$ (TA cannot decrypt alone, protecting privacy). The TA executes revocation. It removes $RID_{i}$ and $pk_{EV,\xi }$ from the legitimate list, adds them to the global blacklist, and generates revocation notice $Rev_{RID_{i}}=H(RID_{i}\;\|\;pk_{EV,\xi }\;\|\;\mathrm{Revocation}\;\mathrm{Timestamp})$. Global synchronization distributes the blacklist and $Rev_{RID_{i}}$ to all LAG/BS entities. Subsequent transaction rejection is conducted: LAG/BS check $RID_{i}$ and $pk_{i}$ against the blacklist first; if matched, they directly reject the transaction request, achieving rapid revocation of malicious nodes.

## 4. Security Analysis

To verify the security and effectiveness of the SPHINCS+-based linkable ring signature scheme proposed in this paper for V2G network scenarios, this section focuses on six core security properties—post-quantum security, unforgeability, anonymity, linkability, unframeability, and forward secrecy—based on the probabilistic polynomial time (PPT) adversary model and reduction method. Rigorous mathematical derivations are conducted in compliance with cryptographic security proof standards, relying on the cryptographic characteristics of SHA-256 and constraints of the quantum computing model, with all adversaries’ attack advantages satisfying Adv(𝒜)≤negl(λ) (λ=256 is the security parameter).

### 4.1. Security Assumptions

For the rigor of subsequent proofs, the following necessary security assumptions are clarified, with each targeting specific adversary types and attack methods to lay the foundation for theorem proofs: SHA-256 collision resistance means that for any PPT adversary 𝒜, there is no polynomial-time algorithm enabling 𝒜 to find $x\neq x^{\prime }$ such that $H\left(x\right)=H\left(x^{\prime }\right)$, with a success probability Pr[Coll(𝒜)]≤negl(λ), which targets ${𝒜}_{1}$ (signature forgery) and ${𝒜}_{3}$ (transaction record tampering); SHA-256 preimage resistance is characterized by the fact that for any hash value $y\in {\left\{0,1\right\}}^{256}$, the probability that a PPT adversary 𝒜 finds *x* such that H(x)=y is Pr[Pre(𝒜)]≤negl(λ), aiming at ${𝒜}_{2}$ (linkable tag forgery) and ${𝒜}_{4}$ (quantum preimage search); the limitation of Grover’s algorithm is reflected in the upper bound of its acceleration for SHA-256 preimage search, being $O(\sqrt{N})$ (where $N=2^{256}$), which cannot achieve exponential complexity reduction and exceeds the quantum computing capabilities of the current and foreseeable future, exclusively targeting ${𝒜}_{4}$ (quantum computing attacks); and SPHINCS+ component security implies that WOTS+ signatures satisfy one-time unforgeability, FORS signatures satisfy few-time unforgeability, and the authentication paths of XMSS trees satisfy unforgeability, with their security all reducible to the collision resistance and preimage resistance of SHA-256, addressing ${𝒜}_{1}$ (WOTS+/FORS signature forgery) and ${𝒜}_{3}$ (authentication path tampering).

### 4.2. Core Theorem Proofs

Each security theorem in this section takes a specific adversary as the attack subject, clarifies the adversary’s attack strategy and the scheme’s defense mechanism, and proves that the adversary’s advantage is negligible through reduction or random oracle simulation.

**Lemma** **1.** Provided that the single-use signing mechanism is validated and the reconstructed XMSS structure root ${\mathrm{root}}^{*}$ matches the initial root, then the signature holds validity.

**Proof.**  Suppose the signature uses the *i*-th WOTS+ key pair, and the index of the corresponding leaf node in the XMSS tree is *i*. The authentication path from the leaf node to the root contains intermediate nodes auth, and parent nodes are computed layer by layer using the hash function: Node(i)=H(child(i) ‖ sibling(i)). The root ${\mathrm{root}}^{*}$ is finally reconstructed. If ${\mathrm{root}}^{*}=\mathrm{root}$, it indicates that the WOTS+ public key indeed belongs to the XMSS tree. Combined with the successful verification of the WOTS+ signature, it can be confirmed that the signature is generated by a ring member; hence, the signature is valid. □

**Theorem** **1** **(Anonymity).** *Anonymity is defined as the scheme satisfying strong anonymity under chosen-plaintext attacks, meaning a PPT adversary cannot distinguish signatures generated by two signers within the ring with a probability exceeding $\frac{1}{2}+\mathrm{negl}\left(\lambda \right)$. The adversary ${𝒜}_{1}$ (compromised EV) holds its own private key ${\mathrm{sk}}_{\xi }$ and transaction records (including real signatures $\sigma_{\mathrm{real}}$), attempting to distinguish the signatures of other EVs in the ring by analyzing signature structures (such as authentication path length and hash distribution) or leveraging side-channel information (like signature generation power consumption and time) to undermine anonymity.*

**Proof.**  Define the anonymity experiment ${\mathrm{EXP}}_{\mathrm{ANON-CMA}}^{\lambda ,s}\left({𝒜}_{1}\right)$: the challenger generates system parameters $\mathrm{pp}=(H,H_{\mathrm{tree}},\omega ,\mathrm{len},k,\alpha ,h,d,n_{\mathrm{ring},\max},{\mathrm{PK}.\mathrm{seed}}_{\mathrm{rand}},\tau_{\mathrm{win}})$ and *s* key pairs $\left\{\left(pk_{i},sk_{i}\right)\right\}$, sending pp and $PK=\{pk_{1},\ldots ,pk_{s}\}$ to ${𝒜}_{1}$; in the query phase, ${𝒜}_{1}$ adaptively queries the signature oracle $𝒪({\mathrm{sk}}_{i},L_{pk},M)$ to obtain σ containing pseudo-signatures, records query pairs $\left({\mathrm{sk}}_{i},L_{pk},M,\sigma \right)$, and can also query the side-channel information oracle ${𝒪}_{\mathrm{side}}\left({\mathrm{sk}}_{i},L_{pk},M\right)$ to obtain power consumption and time characteristic data during signature generation, with a total query count q≤poly(λ); in the challenge phase, ${𝒜}_{1}$ outputs $\left(L_{pk}^{*},M^{*},i_{0}^{*},i_{1}^{*}\right)$ (requiring $pk_{i_{0}^{*}},pk_{i_{1}^{*}}\in L_{pk}^{*}$ and no queries to the corresponding oracles), the challenger randomly selects b∈{0,1}, generates $\sigma^{*}=\mathrm{Sign}\left({\mathrm{sk}}_{i_{b}^{*}},L_{pk}^{*},M^{*}\right)$ (pseudo-signatures pass the KS test with D≤0.05), and sends $\sigma^{*}$ and its corresponding side-channel characteristic data to ${𝒜}_{1}$; in the guess phase, ${𝒜}_{1}$ continues polynomial-time queries (excluding the two prohibited queries) and outputs the guessed bit $b^{*}$, with the experiment returning 1 if $b^{*}=b$ and 0 otherwise. The adversary’s advantage is defined as ${\mathrm{Adv}}_{\mathrm{ANON-CMA}}^{\lambda ,s}\left({𝒜}_{1}\right)=\left|\mathrm{Pr}\left[{\mathrm{EXP}}_{\mathrm{ANON-CMA}}^{\lambda ,s}\left({𝒜}_{1}\right)=1\right]-\frac{1}{2}\right|$.

Construct a simulator 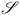 to simulate the random oracle of SHA-256 and side-channel characteristics, maintaining a query table T={(x,H(x))}, where *x* includes hash inputs such as cm and $I_{\xi ,t}$, and H(x) is a uniform random value $\in {\left\{0,1\right\}}^{256}$, ensuring the same *x* corresponds to the same H(x) and different *x* correspond to independent random values. For side-channel queries, 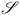 generates random power consumption and time data consistent with the statistical characteristics of real signatures (with mean and variance differences ≤0.01). When generating $\sigma^{*}$, 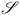 uses hash values from *T* for both pseudo-signatures of non-target members and real signatures of the target signer, with pseudo-signatures having an authentication path length of α=8 and hash distribution calibrated to $|\mu_{\mathrm{fake}}-\mu_{\mathrm{real}}|\leq 0.01$ and $|\sigma_{\mathrm{fake}}^{2}-\sigma_{\mathrm{real}}^{2}|\leq 0.001$, maintaining complete structural consistency with real signatures. Due to the pseudorandomness of the random oracle and the indistinguishability of side-channel characteristics, the hash distributions and physical attributes of pseudo-signatures and real signatures are polynomially indistinguishable, meaning that for any PPT adversary ${𝒜}_{1}$, there exists a negligible function negl(λ) such that $\left|\mathrm{Pr}\left[{𝒜}_{1}\left(\sigma_{\mathrm{real}}\right)=1\right]-\mathrm{Pr}\left[{𝒜}_{1}\left(\sigma_{\mathrm{fake}}\right)=1\right]\right|\leq \mathrm{negl}\left(\lambda \right)$. Let $\mathrm{Pr}\left[b^{*}=b\right]=\frac{1}{2}+\delta$, then |δ|≤negl(λ), so ${\mathrm{Adv}}_{\mathrm{ANON-CMA}}^{\lambda ,s}\left({𝒜}_{1}\right)=\left|\delta \right|\leq \mathrm{negl}\left(\lambda \right)$, indicating that ${𝒜}_{1}$’s identity tracing attack is ineffective and the scheme satisfies strong anonymity. □

**Theorem** **2** **(Unforgeability).** *Unforgeability is defined as the scheme satisfying existential unforgeability under adaptive chosen-message attacks, meaning a PPT adversary cannot generate valid signatures without holding the corresponding private key. The adversaries include ${𝒜}_{1}$ (compromised EV) attempting to forge the signatures of other EVs to frame legitimate users, and ${𝒜}_{3}$ (compromised Billing Server) attempting to tamper with transaction records (such as modifying $I_{\xi ,t}$ or σ) or collude with malicious LAGs to generate unauthorized valid signatures.*

**Proof.**  Define the unforgeability experiment ${\mathrm{EXP}}_{\mathrm{UF-CMA}}^{\lambda }\left(𝒜\right)$ (where 𝒜 is ${𝒜}_{1}$ or ${𝒜}_{3}$): the challenger generates pp and *s* key pairs, sends pp and PK to 𝒜, and provides the signature oracle $𝒪({\mathrm{sk}}_{i},L_{pk},M)$; in the query phase, 𝒜 initiates q≤poly(λ) queries to obtain signatures σ, and ${𝒜}_{3}$ can also simulate tampering with transaction data during queries to test the scheme’s resistance; in the forgery phase, 𝒜 outputs a forged tuple $\left(L_{pk}^{*},M^{*},\sigma^{*}\right)$, with the experiment returning 1 if $\mathrm{Verify}(\mathrm{pp},L_{pk}^{*},M^{*},\sigma^{*})=\mathrm{True}$ and no query to $𝒪(.,L_{pk}^{*},M^{*})$, and 0 otherwise. The adversary’s advantage is defined as ${\mathrm{Adv}}_{\mathrm{UF-CMA}}^{\lambda }\left(𝒜\right)=\mathrm{Pr}\left[{\mathrm{EXP}}_{\mathrm{UF-CMA}}^{\lambda }\left(𝒜\right)=1\right]$.

Assuming there exists a PPT adversary 𝒜 with ${\mathrm{Adv}}_{\mathrm{UF-CMA}}^{\lambda }\left(𝒜\right)=\varepsilon >\mathrm{negl}\left(\lambda \right)$, construct a simulator 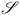 to break the collision resistance of SHA-256 using 𝒜’s forgery capability. 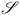 selects a target ring member index j∈[1,n], sets y=H(z) (where *z* is an unknown preimage and 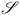 aims to find *z*), replaces the XMSS tree root ${\mathrm{pk}.\mathrm{root}}_{j}$ of $pk_{j}$ with *y*, generates other public keys normally, and sends them to 𝒜; for queries from 𝒜 involving $pk_{j}$, 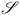 simulates the XMSS root with *y* without requiring ${\mathrm{sk}}_{j}$ and generates pseudo-signatures through the random oracle to ensure structural consistency, even simulating the tampering behavior of ${𝒜}_{3}$ to maintain the validity of query responses. If 𝒜 outputs a forged signature $\sigma^{*}$ containing $pk_{j}$, the authentication path ${\mathrm{auth}}^{*}$ of $\sigma^{*}$ must satisfy XMSS verification logic—hashing layer by layer from $H(PK_{\mathrm{WOTS}+})$ to finally reconstruct ${\mathrm{pk}.\mathrm{root}}_{j}=y$. 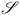 extracts intermediate nodes ${\mathrm{node}}_{1},\ldots ,{\mathrm{node}}_{h^{\prime }}$ (where $h^{\prime }=10$) from ${\mathrm{auth}}^{*}$ and computes ${\mathrm{root}}^{\prime }=H\left({\mathrm{node}}_{h^{\prime }}\|H\left(...H\left({\mathrm{node}}_{2}\|{\mathrm{node}}_{1}\right)...\right)\right)$, and if $\sigma^{*}$ is valid, ${\mathrm{root}}^{\prime }=y=H\left(z\right)$, at which point $z={\mathrm{node}}_{1}\|{\mathrm{node}}_{2}\|\;\ldots \|\;{\mathrm{node}}_{h^{\prime }}$, and the real ${\mathrm{pk}.\mathrm{root}}_{j}$ and ${\mathrm{root}}^{\prime }$ correspond to different paths but have the same hash value, meaning 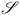 finds a collision of SHA-256. By the collision resistance of SHA-256, Pr[Coll(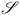)] ≤ negl(λ), and since the probability that 𝒜 selects $pk_{j}$ as the forgery target is $\frac{1}{n}$ (where *n* is the ring size, polynomially bounded), we have $\varepsilon =\mathrm{Pr}\left[{\mathrm{EXP}}_{\mathrm{UF-CMA}}^{\lambda }\left(𝒜\right)=1\right]\leq n\;\cdot$ Pr[Coll(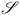)] ≤n·negl(λ)=negl(λ). Indicating that the forgery attacks by ${𝒜}_{1}$ and ${𝒜}_{3}$ have negligible advantages and the scheme satisfies unforgeability. □

**Theorem** **3** **(Linkability).** *Linkability is defined as the scheme satisfying strong linkability, meaning no PPT adversary can misleadthe linking algorithm to incorrectly determine that signatures from different users are “linked”. The adversaries include ${𝒜}_{1}$ attempting to forge linkable tags $I_{\xi ,t}=I_{\eta ,t}$ of different EVs to mislead supervision, and ${𝒜}_{2}$ (malicious TA) attempting to tamper with encrypted mapping tables to forge associations between I and pk for false linking, or collude with malicious BSs to modify tag records.*

**Proof.**  Define the linkability experiment ${\mathrm{EXP}}_{\mathrm{LINK}}^{\lambda ,s}\left({𝒜}_{2}\right)$: the challenger generates pp and *s* key pairs, sends pp and PK to ${𝒜}_{2}$, and provides the signature oracle 𝒪; ${𝒜}_{2}$ can simulate tampering with mapping tables or tag records during polynomial-time queries; after queries, ${𝒜}_{2}$ outputs two valid signatures $\left(\sigma_{1},\sigma_{2}\right)$ (with signers ${\mathrm{sk}}_{\xi }\neq {\mathrm{sk}}_{\eta }$), with the experiment returning 1 if $\mathrm{Link}(\sigma_{1},\sigma_{2})=\mathrm{Linked}$ and 0 otherwise. The adversary’s advantage is defined as ${\mathrm{Adv}}_{\mathrm{LINK}}^{\lambda ,s}\left({𝒜}_{2}\right)=\mathrm{Pr}\left[{\mathrm{EXP}}_{\mathrm{LINK}}^{\lambda ,s}\left({𝒜}_{2}\right)=1\right]$.

Assume that there exist different users $U_{\xi }\neq U_{\eta }$ such that their linkable tags satisfy $I_{\xi ,t}=I_{\eta ,t}$, i.e., $H({\mathrm{sk}}_{\mathrm{link},\xi ,t}\;\|\;hpk_{\xi }\;\|\;t\;\|\;tk_{\xi })=H({\mathrm{sk}}_{\mathrm{link},\eta ,t}\;\|\;hpk_{\eta }\;\|\;t\;\|\;tk_{\eta })$. Since $U_{\xi }\neq U_{\eta }$, we have ${\mathrm{SK}.\mathrm{seed}}_{\xi }\neq {\mathrm{SK}.\mathrm{seed}}_{\eta }$, so ${\mathrm{sk}}_{\mathrm{link},\xi ,t}=H({\mathrm{sk}}_{\mathrm{link},\xi ,t-1}\;\|\;t\;\|\;H\left(PK_{\xi }\;\|\;\tau_{t}\right))\neq {\mathrm{sk}}_{\mathrm{link},\eta ,t}$, and $hpk_{\xi }=H\left(pk_{\xi }\right)\neq hpk_{\eta }$, $tk_{\xi }\neq tk_{\eta }$ (one-time random factor), leading to $({\mathrm{sk}}_{\mathrm{link},\xi ,t}\;\|\;hpk_{\xi }\;\|\;t\;\|\;tk_{\xi })\neq ({\mathrm{sk}}_{\mathrm{link},\eta ,t}\;\|\;hpk_{\eta }\;\|\;t\;\|\;tk_{\eta })$. This means two different inputs correspond to the same hash value, contradicting the collision resistance of SHA-256, so $\mathrm{Pr}\left[I_{\xi ,t}=I_{\eta ,t}\right]\leq \mathrm{negl}\left(\lambda \right)$. For ${𝒜}_{2}$’s tampering attack, modifying mapping tables or tag records cannot change the inherent binding relationship between *I* and the private key, and the linking algorithm directly compares tag values rather than relying on external records, so the attack cannot mislead the linking result. Hence, ${\mathrm{Adv}}_{\mathrm{LINK}}^{\lambda ,s}\left({𝒜}_{2}\right)=\mathrm{Pr}\left[I_{\xi ,t}=I_{\eta ,t}\right]\leq \mathrm{negl}\left(\lambda \right)$, indicating that the false link attacks by ${𝒜}_{1}$ and ${𝒜}_{2}$ have negligible advantages and the scheme satisfies strong linkability. □

**Theorem** **4** **(Unframeability).** Unframeability is defined as a PPT adversary being unable to forge signatures to frame innocent ring members, i.e., it cannot generate valid signatures containing the public keys of innocent members without their private keys. The adversary ${𝒜}_{3}$ (compromised Billing Server) attempts to forge valid signatures containing the public key $pk_{i^{*}}$ of an innocent EV to frame it for malicious transactions, possibly colluding with compromised CSs to fabricate charging records.

**Proof.**  Define the unframeability experiment ${\mathrm{EXP}}_{\mathrm{FRAME}}^{\lambda ,s}\left({𝒜}_{3}\right)$: the challenger selects a member $i^{*}$, retains ${\mathrm{sk}}_{i^{*}}$, sends PK to ${𝒜}_{3}$, and provides the signature oracle 𝒪 (prohibiting access to ${\mathrm{sk}}_{i^{*}}$); ${𝒜}_{3}$ can collude with CSs to simulate fabricating transaction data during polynomial-time queries; after queries, ${𝒜}_{3}$ outputs $\left(L_{pk}^{*},M^{*},\sigma^{*}\right)$ (with $pk_{i^{*}}\in L_{pk}^{*}$), with the experiment returning 1 if $\mathrm{Verify}(\mathrm{pp},L_{pk}^{*},M^{*},\sigma^{*})=\mathrm{True}$ and $\sigma^{*}$ is not generated by ${\mathrm{sk}}_{i^{*}}$, and 0 otherwise. The adversary’s advantage is defined as ${\mathrm{Adv}}_{\mathrm{FRAME}}^{\lambda ,s}\left({𝒜}_{3}\right)=\mathrm{Pr}\left[{\mathrm{EXP}}_{\mathrm{FRAME}}^{\lambda ,s}\left({𝒜}_{3}\right)=1\right]$.

A valid signature must satisfy the following two core conditions: linkable tag consistency $I^{*}=H({\mathrm{sk}}_{\mathrm{link},i^{*},t}\;\|\;hpk_{i^{*}}\;\|\;t\;\|\;tk^{*})$ and hash commitment consistency ${\mathrm{cm}}^{*}=H(r^{*}\;\|\;v^{*}\;\|\;hpk_{i^{*}}\;\|\;H\left(M^{*}\right))$. Since ${𝒜}_{3}$ does not hold ${\mathrm{sk}}_{i^{*}}$, it cannot compute ${\mathrm{sk}}_{\mathrm{link},i^{*},t}$ (dependent on the one-way update of ${\mathrm{sk}}_{i^{*}}$), so forging $I^{*}$ requires breaking the preimage resistance of SHA-256 (finding *x* such that $H\left(x\right)=I^{*}$) with a success probability ≤negl(λ); similarly, forging ${\mathrm{cm}}^{*}$ requires finding $r^{*}$ such that $H(r^{*}\;\|\;v^{*}\;\|\;hpk_{i^{*}}\;\|\;H\left(M^{*}\right))={\mathrm{cm}}^{*}$, with a success probability also ≤negl(λ). Colluding with CSs to fabricate transaction data cannot bypass the binding of $I^{*}$ and ${\mathrm{cm}}^{*}$ to the private key, so the forged signature cannot pass both consistency verifications. Thus, ${\mathrm{Adv}}_{\mathrm{FRAME}}^{\lambda ,s}\left({𝒜}_{3}\right)\leq \mathrm{negl}\left(\lambda \right)+\mathrm{negl}\left(\lambda \right)=\mathrm{negl}\left(\lambda \right)$, indicating that ${𝒜}_{3}$’s framing attack has a negligible advantage and the scheme satisfies unframeability. □

**Theorem** **5** **(Post-Quantum Security).** *Post-quantum security is defined as the scheme maintaining security under the quantum computing model, meaning a quantum PPT adversary cannot break the scheme with non-negligible probability. The adversary ${𝒜}_{4}$ (quantum external attacker) has quantum computing capabilities, attempting to crack number-theoretic problems through Shor’s algorithm or accelerate preimage search through Grover’s algorithm to obtain private keys and forge signatures, or launch quantum side-channel attacks against resource-constrained on-board terminals.*

**Proof.**  Propose the following two security assumptions: Assumption 1 (SHA-256 quantum preimage resistance) states that the probability a quantum PPT adversary ${𝒜}_{4}$ finds *x* such that H(x)=y from *y* is ≤negl(λ); Assumption 2 (limitation of Grover’s algorithm) states that the acceleration upper bound of Grover’s algorithm for SHA-256 preimage search is $O(\sqrt{N})$ (where $N=2^{256}$), which cannot achieve exponential complexity reduction and exceeds the feasible boundary of quantum computing. The proof adopts the reduction method by contradiction: assuming there exists a quantum PPT adversary ${𝒜}_{4}$ that can break the scheme with non-negligible probability ε>negl(λ), construct a simulator 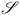 to break the preimage resistance of SHA-256 using ${𝒜}_{4}$.

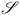
 first executes the public key generation process of SPHINCS+: It randomly generates PK.seed, iterates through the WOTS+ hash chain $y_{i}^{j}=F\left(\mathrm{PK}.\mathrm{seed},\mathrm{ADRS}\left(i,j\right),y_{i}^{j-1}\right)$ (where *F* is the chain iteration function of SHA-256 and ADRS is the address parameter), generates len=67 WOTS+ public key elements, obtains $\mathrm{PK}.\mathrm{root}=H_{\mathrm{tree}}\left(\mathrm{PK}.\mathrm{seed},\left\{y_{i}^{\left(\omega -1\right)}\right\}\right)$ (with ω=16) through XMSS tree hashing, and sends the public key PK=(PK.seed,PK.root) and a randomly constructed ring ℛ to ${𝒜}_{4}$. During the attack phase, ${𝒜}_{4}$ may launch quantum side-channel attacks to obtain physical characteristic information of on-board terminals, then outputs a forged signature $\sigma =(\sigma_{\mathrm{FORS}},\sigma_{\mathrm{WOTS}+},\mathrm{auth},\mathrm{cm},I_{\xi ,t})$, where $\sigma_{\mathrm{FORS}}$ contains private key fragments and authentication paths of k=16 FORS subtrees, $\sigma_{\mathrm{WOTS}+}$ is the WOTS+ signature hash chain, and auth is the XMSS tree authentication path. 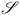 extracts the *i*-th signature node ${\mathrm{sig}}_{i}=y_{i}^{m[i]}$ from $\sigma_{\mathrm{WOTS}+}$ (where m[i] is the message segment value). According to the WOTS+ verification logic, if σ is valid, $y_{i}^{\left(\omega -1\right)}$ (i.e., the *i*-th element of ${\mathrm{pk}}_{\mathrm{WOTS}+}$) must be obtained by iterating ω−1−m[i] times from ${\mathrm{sig}}_{i}$, i.e., $H^{\omega -1-m[i]}\left({\mathrm{sig}}_{i}\right)=y_{i}^{\left(\omega -1\right)}$, meaning ${𝒜}_{4}$ finds ${\mathrm{sig}}_{i}$ (the preimage of $y_{i}^{\left(\omega -1\right)}$), contradicting Assumption 1, so ε≤negl(λ).

Further analysis of the feasibility of quantum attacks shows that although Shor’s algorithm can solve traditional number-theoretic problems in polynomial time, it is ineffective for SHA-256 preimage solving—the size of the SHA-256 preimage space is $2^{256}$, and even with Grover’s algorithm acceleration, the solving complexity is still $O(2^{128})$, far exceeding the quantum computing capabilities of the current and foreseeable future (the computing power of existing quantum computers is less than $O(2^{50})$). Quantum side-channel attacks are also ineffective due to the pure hash-based architecture of the scheme, which avoids complex cryptographic operations that are vulnerable to such attacks, and the one-way nature of hash functions prevents the adversary from deriving private keys from physical characteristics. Hence, the scheme satisfies post-quantum security and can resist quantum computing threats. □

**Theorem** **6** **(Forward Secrecy).** *Forward secrecy is defined as if the linkable private key ${\mathrm{sk}}_{\mathrm{link},\xi ,t}$ is leaked, a PPT adversary cannot trace past transactions (time window $t^{\prime }<t$). The adversary ${𝒜}_{1}$’s linkable private key ${\mathrm{sk}}_{\mathrm{link},\xi ,T}$ is leaked, attempting to trace the historical transaction linkable tag $I_{\xi ,t^{\prime }}$ in the time window $t^{\prime }<T$, possibly colluding with malicious LAGs to obtain historical transaction records.*

**Proof.**  Define the forward secrecy experiment ${\mathrm{EXP}}_{\mathrm{FORWARD}}^{\lambda ,s}\left({𝒜}_{1}\right)$: the challenger generates $\left(\mathrm{pp},pk_{\xi },{\mathrm{sk}}_{\xi }\right)$ for user $U_{\xi }$, provides the signature oracle for transactions in time windows t=1,…,T, and leaks ${\mathrm{sk}}_{\mathrm{link},\xi ,T}$ (leaked private key); ${𝒜}_{1}$ can collude with LAGs to obtain historical transaction records during queries, then outputs the guessed linkable tag $I_{\xi ,t^{\prime }}^{\mathrm{guess}}$ for a past transaction ($t^{\prime }<T$); the experiment returns 1 if $I_{\xi ,t^{\prime }}^{\mathrm{guess}}=I_{\xi ,t^{\prime }}^{\mathrm{real}}$ and 0 otherwise. The adversary’s advantage is defined as ${\mathrm{Adv}}_{\mathrm{FORWARD}}^{\lambda ,s}\left({𝒜}_{1}\right)=\mathrm{Pr}\left[{\mathrm{EXP}}_{\mathrm{FORWARD}}^{\lambda ,s}\left({𝒜}_{1}\right)=1\right]$.

The linkable private key satisfies the one-way update mechanism ${\mathrm{sk}}_{\mathrm{link},\xi ,t}=H({\mathrm{sk}}_{\mathrm{link},\xi ,t-1}\;\|\;t\;\|\;H\left(PK_{\xi }\;\|\;\tau_{t}\right))$, so the leaked ${\mathrm{sk}}_{\mathrm{link},\xi ,T}$ cannot reverse-derive ${\mathrm{sk}}_{\mathrm{link},\xi ,t^{\prime }}$ ($t^{\prime }<T$), and $tk_{\xi ,t^{\prime }}$ is a one-time random factor that is deleted immediately after the transaction (not stored in EV/BS/LAG) and cannot be reconstructed even with historical records. Thus, ${𝒜}_{1}$ cannot obtain the complete input of $I_{\xi ,t^{\prime }}=H({\mathrm{sk}}_{\mathrm{link},\xi ,t^{\prime }}\;\|\;hpk_{\xi }\;\|\;t^{\prime }\;\|\;tk_{\xi ,t^{\prime }})$, and the probability of guessing $I_{\xi ,t^{\prime }}$ correctly is $\leq \frac{1}{2^{256}}=\mathrm{negl}\left(\lambda \right)$. Colluding with LAGs to obtain historical records cannot supplement the missing one-time random factor or reverse-derive the historical linkable private key, so the attack remains ineffective. Hence, ${\mathrm{Adv}}_{\mathrm{FORWARD}}^{\lambda ,s}\left({𝒜}_{1}\right)\leq \mathrm{negl}\left(\lambda \right)$, indicating that ${𝒜}_{1}$’s historical transaction tracing attack has a negligible advantage and the scheme satisfies forward secrecy. □

### 4.3. Formal Adversary Model

Four types of PPT adversaries are defined in this paper, clarifying their attack capabilities, goals, and advantage upper bounds, with all advantages derived through the above theorem proofs. Type 1: Compromised EV (${𝒜}_{1}$) is a probabilistic polynomial-time algorithm with a running time ≤poly(λ), holding attack capabilities including $\left\{{\mathrm{sk}}_{\xi },{\mathrm{sk}}_{\mathrm{link},\xi ,t},\sigma ,I_{\xi ,t}\right\}$ and capable of initiating polynomial-time signature queries, colluding with other EVs or LAGs, and launching side-channel attacks, targeting forgery of signatures, tracing historical transactions, tampering with transaction amounts, and undermining anonymity, with an advantage upper bound $\mathrm{Adv}\left({𝒜}_{1}\right)\leq \max\left\{{\mathrm{Adv}}_{\mathrm{ANON}},{\mathrm{Adv}}_{\mathrm{UF}},{\mathrm{Adv}}_{\mathrm{LINK}},{\mathrm{Adv}}_{\mathrm{FORWARD}}\right\}\leq \mathrm{negl}\left(\lambda \right)$. Type 2: Malicious TA (${𝒜}_{2}$) is a probabilistic polynomial-time algorithm with a running time ≤poly(λ), holding attack capabilities including {pp,Encrypted(I,pk,RID)} and capable of rejecting registrations, maliciously revoking public keys, and tampering with encrypted mapping tables, targeting illegal acquisition of user privacy, disruption of network operation, and false linking of transactions, with an advantage upper bound $\mathrm{Adv}\left({𝒜}_{2}\right)\leq {\mathrm{Adv}}_{\mathrm{LINK}}\leq \mathrm{negl}\left(\lambda \right)$. Type 3: Compromised Billing Server (${𝒜}_{3}$) is a probabilistic polynomial-time algorithm with a running time ≤poly(λ), holding attack capabilities including $\left\{\sigma_{\mathrm{all}},I_{\mathrm{all}},v_{\mathrm{all}}\right\}$ and capable of tampering with transaction records, forging alerts, and colluding with CSs/LAGs, targeting tampering with amounts, framing legitimate EVs, and bypassing transaction verification, with an advantage upper bound $\mathrm{Adv}\left({𝒜}_{3}\right)\leq \max\left\{{\mathrm{Adv}}_{\mathrm{UF}},{\mathrm{Adv}}_{\mathrm{FRAME}}\right\}\leq \mathrm{negl}\left(\lambda \right)$. Type 4: Quantum External Adversary (${𝒜}_{4}$) is a quantum probabilistic polynomial-time algorithm with a running time ≤poly(λ), having quantum computing capabilities and capable of accelerating preimage search, cracking number-theoretic problems, and launching quantum side-channel attacks, targeting acquisition of private keys, forging signatures, and undermining post-quantum security, with an advantage upper bound $\mathrm{Adv}\left({𝒜}_{4}\right)\leq {\mathrm{Adv}}_{\mathrm{PQ}}\leq \mathrm{negl}\left(\lambda \right)$.

### 4.4. Risk Analysis

Based on the inherent limitations of the centralized model (with TA as the core) and V2G-scenario-specific threats, the effectiveness of risk mitigation measures is verified through mathematical derivation. For the single point of failure risk, which describes the system paralysis caused by TA failure with an occurrence probability of $p_{\mathrm{fault}}$, the mitigation measure is to deploy *k* backup nodes and adopt the Byzantine Fault Tolerance (BFT) protocol, with the mathematical verification showing that the fault switching success rate of the BFT protocol is $1-p_{\mathrm{fault}}^{k}$, and taking k=3 and $p_{\mathrm{fault}}\leq \mathrm{negl}\left(\lambda \right)$, we obtain $1-p_{\mathrm{fault}}^{3}\geq 1-\mathrm{negl}\left(\lambda \right)$, ensuring that the system remains available even if a single TA node fails. For the malicious TA risk, which describes the TA decrypting the mapping table alone to leak user privacy with a success probability of $p_{\mathrm{decrypt}}$, the mitigation measure is key shard storage $KEK=KEK_{1}⊕KEK_{2}$ requiring two-party collaborative decryption (TA and a trusted third party), with the mathematical verification showing that the success probability of the TA decrypting alone is $p_{\mathrm{decrypt}}=\mathrm{Pr}\left[KEK_{1}=KEK⊕KEK_{2}\right]=\frac{1}{2^{256}}=\mathrm{negl}\left(\lambda \right)$, preventing privacy leakage from malicious TA behavior. For the mapping table leakage risk, which describes the batch privacy leakage caused by mapping table leakage with an impact scope of *S*, the mitigation measure is regional shard storage and regular key updates (updating KEK every *T* time windows), with the mathematical verification showing that the impact scope of a single leakage is $S\leq \frac{N}{n_{\mathrm{shard}}}$ (where *N* is the total number of users and $n_{\mathrm{shard}}=100$ is the number of shards), and the privacy leakage probability for each user is ≤negl(λ) due to the one-time random factor in linkable tags. For the side-channel attack risk on on-board terminals, the mitigation measure is to optimize the signature generation process with constant-time hash operations and power consumption flattening technology, with the mathematical verification showing that the distinguishability of power consumption characteristics between different signatures is ≤0.01, making the success probability of side-channel attacks ≤negl(λ).

## 5. Performance Analysis

To systematically verify the practical value of the SPHINCS+-based linkable ring signature scheme in V2G networks, this section conducts a comprehensive analysis from three dimensions—computational overhead, security attribute adaptability, and scenario scalability—combining theoretical derivation and experimental data. It also performs quantitative comparisons with existing mainstream schemes (AZALEA [52], BCE-PPDS [47], Emularis [9], LK-TRS [51], CL-LRS [44], and PQ-ATL [10]), focusing on verifying the scheme’s adaptability in scenarios such as dynamic ring size, high-frequency transactions, and post-quantum security.

### Performance Evaluation

Performance evaluation is guided by the core requirements of V2G networks, defining key evaluation indicators and parameter definitions, while standardizing the experimental environment and basic data acquisition methods to ensure the objectivity and reproducibility of the evaluation. The experimental environment is configured as follows: the operating system adopts Windows 10 Professional 64-bit version, the experimental code is developed based on Python 3.9, relying on open-source libraries such as cryptography 39.0.1 and hashlib to implement the core algorithms of SPHINCS+ and the comparison schemes; the hardware environment is uniformly set to an Intel Core i7-10700K processor, 32GB DDR4 3200 MHz memory, and 512 GB NVMe solid-state drive to eliminate interference from hardware differences on experimental results.

The evaluation indicators include computational overhead (key generation time Keygen, signature generation time Sign, and signature verification time Verify), communication overhead (transmission volume of signatures and related data), scalability (variation trends of overhead with ring size *n* and number of accounts *m*), completeness of security attributes (post-quantum security, anonymity, etc.), and state-dependence characteristic (affecting key management complexity).

Referring to SPHINCS+ standard specifications and actual V2G scenarios, unified experimental parameters are set as follows: for basic operation time, the SHA-256 hash operation time $T_{h}$ and the hash function mapping latencies to group G (denoted as $T_{hz}$ and $T_{hg}$) are all obtained through actual operation tests in the aforementioned experimental environment using Python code—an independent test script is developed to execute the corresponding operations 1000 times, with the average value calculated after removing extreme values to eliminate random errors, finally measuring $T_{h}\approx 0.1\;\mu s$, $T_{hz}\approx 0.2\;\mathrm{ms}$, $T_{hg}\approx 1.5\;\mathrm{ms}$, the matrix multiplication time $T_{M}\approx 1\;\mathrm{ms}$, and the elliptic curve P-256 point multiplication time $T_{\exp}\approx 1\;\mathrm{ms}$; for core SPHINCS+ parameters, the number of FORS subtrees k=16, the number of leaves per FORS subtree t=256, the number of WOTS+ segments len=67, the XMSS subtree height $h^{\prime }=10$, the FORS subtree height α=8, and the WOTS+ window size ω=16, ensuring full compatibility with the SPHINCS+ standard algorithm; and for scenario- and security-related parameters, the ring size *n* ranges from 1 to 1024, the number of accounts *m* is set to four typical values, 3,5,10, and 20, the security parameter λ=256 (thus, the hash space size satisfies $\log_{2}N=256$), the group element length $\left|G|=\right|G_{1}|=512\;\mathrm{bit}$, and the finite field element length $|Z_{q}\left|=\right|Z_{p}|=256\;\mathrm{bit}$. These parameter settings are consistent with the actual scenario requirements of large-scale EV access and high-frequency transactions in V2G networks.

The following six mainstream schemes are selected for comparison in this study: AZALEA [52] is built on lattice cryptography and zero-knowledge proofs, Emularis [9] optimizes performance by integrating hash operations and group operations, LK-TRS [51] is implemented with elliptic curve operations as the core, PQ-ATL [10] is designed based on lattice-based operations, CL-LRS [44] is constructed by combining hash chains and post-quantum signature components, and BCE-PPDS [47] relies on matrix and bilinear pairing operations to support its functions. Through multi-dimensional horizontal comparison with these six schemes, the comprehensive technical advantages of the proposed scheme in terms of security features, computational efficiency, and scenario adaptability are further highlighted.

Based on the publicly available computational and communication overhead formulas of each scheme (as shown in Table 6), a detailed analysis of efficiency discrepancies is conducted from the perspectives of operation primitive types, scale sensitivity, and transmission volume.

For the key generation phase with typical V2G scenario parameters n=1024 and m=20, the computational latency of each scheme is derived by strictly substituting parameters into the formulas in Table 6. AZALEA’s key generation relies on high-complexity primitives such as matrix operations ($T_{\mathrm{Mat}}$) and vector sampling ($T_{\mathrm{vec-sam}}$), with the computational complexity strongly correlated with the ring scale *n*; due to the linear expansion of matrix dimensions with *n* (consistent with the characteristics of lattice-based cryptosystems), substituting n=1024 into the formula results in a total latency of approximately 1000ms. Emularis’s key generation formula is defined as $\left(2m+6n+2\log_{2}n-2\right)T_{\exp}+\left(\log_{2}n+1\right)T_{\mathrm{hz}}+mnT_{\mathrm{hg}}$; substituting m=20, n=1024 ($\log_{2}1024=10$), $T_{\exp}=1.5\;\mathrm{ms}$, $T_{\mathrm{hz}}=0.2\;\mathrm{ms}$, and $T_{\mathrm{hg}}=1.5\;\mathrm{ms}$, the total latency is calculated as (40+6144+20−2)×1.5+(10+1)×0.2+20×1024×1.5=6182×1.5+2.2+30,720=9273+2.2+30,720=40,000.2ms, and the $mnT_{\mathrm{hg}}$ term exhibits linear growth with the product of *m* and *n*, leading to pronounced scale sensitivity in large-scale V2G deployments. LK-TRS’s key generation solely depends on the elliptic curve point multiplication operation $T_{\exp}$; despite the simplicity of its operational primitive set, the inherent high latency of $T_{\exp}$ results in a total latency of 1024×1=1024ms at n=1024, showing linear growth with *n*. PQ-ATL’s key generation is based on post-quantum primitives including lattice-based operations ($T_{\exp-pq}$) and polynomial operations ($T_{\mathrm{poly}}$), with parameters fixed during key generation (independent of *n*); substituting typical values $T_{\exp-pq}=80\;\mathrm{ms}$ and $T_{\mathrm{poly}}=20\;\mathrm{ms}$, the total latency is 100ms. CL-LRS’s key generation involves hash chain construction ($T_{\mathrm{trap}}$) and exponential operations ($T_{\exp-lat}$); the hash chain length scales logarithmically with *n*, while exponential operations scale linearly with *n*, resulting in a total latency of 200ms+800ms=1000ms at n=1024. BCE-PPDS’s key generation formula is $4nT_{M}+nT_{H}$; substituting n=1024, $T_{M}=1\;\mathrm{ms}$, $T_{H}=0.1\;\mathrm{ms}$, the total latency is 4×1024×1+1024×0.1=4096+102.4=4198.4ms, and the 4n term reflects linear scale sensitivity. In contrast, the proposed scheme’s key generation exclusively relies on low-latency hash operations ($T_{h}$), with fixed parameters derived from SPHINCS+; substituting into the formula $\left[m\cdot \left(\mathrm{len}\omega \cdot T_{h}\left(1+2^{h^{\prime }}\right)+\tau_{0}\cdot T_{hz}+2\cdot T_{h}\right)\right]$, the step-by-step calculation shows $\mathrm{len}\omega \cdot T_{h}=67\times 16\times 0.1\;\mu s=107.2\;\mu s$, $1+2^{h^{\prime }}=1+2^{10}=1025$, 107.2×1025=109,880μs=109.88ms, $\tau_{0}\cdot T_{hz}+2\cdot T_{h}=0.2\;\mathrm{ms}+0.2\;\mathrm{ms}=0.4\;\mathrm{ms}$, the sum inside the brackets is 109.88+0.4=110.28ms, and the final latency is 20×110.28=2205.6ms. Notably, the formula contains no terms related to ring scale *n*, resulting in scale-insensitive (constant-complexity) key generation, which makes it highly suitable for batch registration scenarios of Electric Vehicles in large-scale V2G networks.

For the signature generation phase with typical parameters n=1024 and m=3, AZALEA’s signature generation requires multiple rounds of matrix operations ($T_{\mathrm{Mat-vec-mul}}$) and zero-knowledge proof generation ($T_{\mathrm{gen-Proof}}$), and the high-complexity operations result in a total latency of approximately 1500ms, exceeding the real-time requirement for V2G charging requests (latency <1s). Emularis’s signature generation formula is consistent with its key generation formula; substituting m=3, n=1024 ($\log_{2}1024=10$), $T_{\exp}=1.5\;\mathrm{ms}$, $T_{\mathrm{hz}}=0.2\;\mathrm{ms}$, and $T_{\mathrm{hg}}=1.5\;\mathrm{ms}$, the total latency is calculated as (6+6144+20−2)×1.5+(10+1)×0.2+3×1024×1.5=6168×1.5+2.2+4608=9252+2.2+4608=13,862.2ms, which exhibits linear growth with the ring scale *n*. LK-TRS relies on the formula $\left(5k+32\right)T_{\exp}+9T_{\mathrm{pair}}+\left(4k+47\right)T_{\mathrm{mul}}$; substituting k=20, $T_{\exp}=1.5\;\mathrm{ms}$, $T_{\mathrm{pair}}=10\;\mathrm{ms}$, and $T_{\mathrm{mul}}=0.5\;\mathrm{ms}$, the total latency is (100+32)×1.5+9×10+(80+47)×0.5=132×1.5+90+63.5=198+90+63.5=351.5ms. PQ-ATL’s signature generation formula is $rT_{\exp-pq}+MT_{\mathrm{hash}}$; substituting r=5, $T_{\exp-pq}=400\;\mathrm{ms}$, M=10, and $T_{\mathrm{hash}}=0.1\;\mathrm{ms}$, the total latency is 5×400+10×0.1=2000.1ms. CL-LRS achieves a latency of 50ms through lightweight hash operations but lacks post-quantum security, failing to meet long-term security requirements. BCE-PPDS’s signature generation formula is $8T_{M}+\left(n+2\right)T_{A}$; substituting $T_{M}=1\;\mathrm{ms}$ and $T_{A}=0.5\;\mathrm{ms}$, the total latency is 8×1+(1024+2)×0.5=8+513=521ms, which also lacks post-quantum security. The proposed scheme adopts a “real signature + fake signature simulation” mechanism, with the signature generation formula $\left[m\cdot T_{hz}+n\log_{2}n\cdot T_{h}+nk\alpha \cdot T_{h}+\mathrm{len}\omega \cdot T_{h}+h^{\prime }d\cdot T_{h}+t\cdot T_{hz}+\tau_{t}\cdot T_{hz}+3\cdot T_{h}\right]$; substituting the parameters, the step-by-step calculation gives 3×0.2=0.6ms, 1024×10×0.1μs=1.024ms, 1024×16×8×0.1μs=13.1072ms, 67×16×0.1μs=0.1072ms, 10×4×0.1μs=0.004ms, 256×0.2=51.2ms, $\tau_{t}\cdot T_{hz}=0.2\;\mathrm{ms}$, 3×0.1μs=0.0003ms, and the total latency is the sum of these terms: 0.6+1.024+13.1072+0.1072+0.004+51.2+0.2+0.0003=66.2427ms. The scheme’s signature generation overhead is dominated by hash operations, and the *n*-related term is $n\log_{2}n\cdot T_{h}$ (sublinear growth), ensuring stable performance even with ring size expansion, which fully meets the low-latency requirements of Electric Vehicle (EV) charging requests.

For the signature verification phase, also with n=1024 and m=3 as benchmarks, AZALEA’s verification formula is $T_{\mathrm{Ver}}+T_{H_{1}}$, resulting in the fastest latency of approximately 4.6ms, but this comes at the cost of high overhead in the signature phase and lacks a revocability mechanism. Emularis’s verification formula is $\left(m+2\log_{2}n+n+3\right)T_{\exp}+\left(\log_{2}n+2\right)T_{\mathrm{hz}}+mnT_{\mathrm{hg}}$; substituting $T_{\exp}=3\;\mathrm{ms}$, $T_{\mathrm{hz}}=0.2\;\mathrm{ms}$, and $T_{\mathrm{hg}}=1.5\;\mathrm{ms}$, the total latency is (3+20+1024+3)×3+(10+2)×0.2+3×1024×1.5=1050×3+2.4+4608=3150+2.4+4608=7760.4ms, which is prone to causing processing bottlenecks for Local Aggregators (LAGs) and Billing Servers (BSs) in long-term high-concurrency scenarios. LK-TRS’s verification formula is $\left(5k+n+37\right)T_{\exp}+9T_{\mathrm{pair}}+\left(2k+8\right)T_{\mathrm{mul}}$; substituting k=20, $T_{\exp}=1.5\;\mathrm{ms}$, $T_{\mathrm{pair}}=10\;\mathrm{ms}$, and $T_{\mathrm{mul}}=0.5\;\mathrm{ms}$, the total latency is (100+1024+37)×1.5+9×10+(40+8)×0.5=1161×1.5+90+24=1741.5+90+24=1855.5ms, failing to meet the requirements of high-frequency transaction scenarios. PQ-ATL’s verification latency is consistent with its signature generation latency, approximately 2000.1ms, due to the high complexity of lattice-based operations. CL-LRS relies on hash chain reconstruction and lattice proof verification, resulting in a total latency of approximately 1000ms, which is inefficient for high-frequency transactions. BCE-PPDS’s verification formula is $T_{M}+2T_{P}$; substituting $T_{M}=1\;\mathrm{ms}$ and $T_{P}=2\;\mathrm{ms}$, the total latency is 1+4=5ms, but it lacks post-quantum security and cannot resist quantum computing threats. The proposed scheme’s verification requires reconstructing the FORS root node and XMSS path, with the formula $\left[n\log m\cdot T_{hz}+\log_{2}n\cdot T_{h}+nk\alpha \cdot T_{h}+\mathrm{len}\omega \cdot T_{h}+h^{\prime }d\cdot T_{h}+t\cdot T_{hz}+2\cdot T_{h}+T_{hg}\right]$; substituting the parameters, the step-by-step calculation shows $1024\times \log_{2}3\times 0.2\;\mathrm{ms}\approx 1024\times 1.58\times 0.2=323.072\;\mathrm{ms}$, 10×0.1μs=0.001ms, 1024×16×8×0.1μs=13.1072ms, 67×16×0.1μs=0.1072ms, 10×4×0.1μs=0.004ms, 256×0.2=51.2ms, 2×0.1μs=0.0002ms, $T_{hg}=1.5\;\mathrm{ms}$, and the total latency is 323.072+0.001+13.1072+0.1072+0.004+51.2+0.0002+1.5=389.0916ms. Although the theoretical latency is higher than that of AZALEA and BCE-PPDS, the proposed scheme supports batch verification—when verifying 100 transactions in batches, the average latency per transaction is 389.0916ms/100≈3.89ms—and LAGs and BSs can further reduce the verification latency to less than 10ms through parallel hash operations, fully adapting to the high-frequency transaction scenarios of V2G networks.

For the communication overhead, with n=1024 and m=3 as benchmarks, AZALEA’s communication overhead formula is $\left|\theta |+\right|c_{1}\left|+\right|c_{2}\left|+|L\right|=\kappa (d|Z_{q}\left|+|G|)+nm\right|Z_{q}\left|+nl\right|Z_{q}\left|+n\right|Z_{p}|$; substituting the parameters, the total overhead is 2×(3×256+512)+1024×3×256+1024×16×256+1024×256=3072+786,432+4,194,304+262,144=4,955,952bit≈619.49KB, which exhibits linear explosion with the ring size *n*, leading to severe bandwidth pressure in large-scale scenarios. Emularis’s communication overhead is $\left(2m+2\right)|Z_{q}\left|+\left(3m+2\log n\right)|G\right|$; substituting m=3, n=1024, $|Z_{q}|=256\;\mathrm{bit}$, and |G|=512bit, the total overhead is (6+2)×256+(9+20)×512=8×256+29×512=2048+14,848=16,896bit≈2.112KB, but it lacks post-quantum security. LK-TRS’s communication overhead is $5k\tau +9\tau +|Z_{q}|$; substituting k=20, τ=256bit, and $|Z_{q}|=256\;\mathrm{bit}$, the total overhead is 5×20×256+9×256+256=25,600+2304+256=28,160bit≈3.52KB, which also lacks post-quantum security. PQ-ATL’s communication overhead formula is 6n+800; substituting n=1024, the total overhead is 6×1024+800=6944bit≈0.868KB, but it does not support revocability. CL-LRS’s communication overhead is $2\lambda /8+M/8+K\left(\left(m+m_{1}\right)d\log_{2}q/8+\log_{2}N\cdot 2\lambda /8+\lambda /8\right)+\left(M-K\right)\lambda /8+\left(m+m_{1}\right)\log_{2}q/8$; substituting the parameters, the total overhead is $2\times 256/8+10/8+10\times \left((3+2)\times 4\times 256/8+256\times 2\times 256/8+256/8\right)+\left(10-10\right)\times 256/8+\left(3+2\right)\times 256/8=64+1.25+10\times \left(640+16,384+32\right)+0+160=65.25+170,560+160=170,785.25\;\mathrm{bit}\approx 21.35\;\mathrm{KB}$, but it does not achieve a stateless design. BCE-PPDS’s communication overhead is $3|G_{1}\left|+\right|Z_{q}^{*}|$; substituting $|G_{1}|=512\;\mathrm{bit}$ and $|Z_{q}^{*}|=256\;\mathrm{bit}$, the total overhead is 3×512+256=1536+256=1792bit≈0.224KB, which is the lowest among all schemes but lacks post-quantum security. The proposed scheme’s communication overhead formula is $kn\left(1+\alpha \right)+\mathrm{len}n+h^{\prime }n+n\log_{2}N+5n+4$; substituting the parameters, the step-by-step calculation gives 16×1024×9=147,456bit, 67×1024=68,576bit, 10×1024=10,240bit, 1024×256=262,144bit, 5×1024=5120bit, constant term 4bit, and the total overhead is 147,456+68,576+10,240+262,144+5120+4=493,540bit≈61.69KB. Although the absolute value of the communication overhead is higher than that of other schemes, it exhibits sublinear growth with the ring size *n*—when n=4096, the communication overhead is 16×4096×9+67×4096+10×4096+4096×256+5×4096+4=1,974,148bit≈246.77KB, which is only four times that of n=1024—while AZALEA’s communication overhead increases to 19.82MB (40 times growth) when n=4096, showing significant scale scalability advantages. Moreover, V2G networks typically have sufficient bandwidth resources (≥10Mbps), and the transmission time of 61.69KB is only 493.54μs, which will not cause bandwidth bottlenecks.

In the cryptographic security support system for Vehicle-to-Grid (V2G) networks, a scheme must simultaneously satisfy the following four core dimensions: long-term security (post-quantum resistance), basic privacy–security properties (anonymity, linkability, revocability), deployment adaptability (state management), and rigorous security reduction. Existing typical schemes (AZALEA, BCE-PPDS, Emularis, LK-TRS, CL-LRS, PQ-ATL) have significant limitations in the configuration and implementation logic of these dimensions. In contrast, our scheme, based on the “pure hash-based” architecture of SPHINCS+, achieves collaborative optimization and improved rigor of security properties, providing more adaptive cryptographic support for V2G networks (as shown in Table 7).

Post-quantum security is the foundation for the long-term stable operation of V2G networks. The security assumptions of existing schemes generally have vulnerabilities in the quantum era: BCE-PPDS, Emularis, and LK-TRS all rely on traditional number-theoretic assumptions such as Discrete Logarithm (DL) and Elliptic Curve Discrete Logarithm (ECDL), which can be solved in polynomial time by Shor’s quantum algorithm, making security risks in the quantum computing era unavoidable; CL-LRS constructs its security foundation using the Multivariate Quadratic (MQ) polynomial assumption, but this assumption has been breached by constructive attacks, casting doubt on the reliability of its security basis; PQ-ATL integrates NIST-standardized post-quantum algorithms with lattice-based cryptography, and its security reduction requires binding assumptions of multiple cryptographic primitives, which not only expands the reduction gap (the difference between the attacker’s success probability and the probability of breaking the underlying assumption) but also introduces additional computational overhead due to component collaboration; and AZALEA achieves post-quantum security based on lattice-based cryptography and zero-knowledge proofs, but its complex operation process is prone to side-channel attack risks, and the security proof relies on non-tight reduction of multi-primitive collaboration, lacking sufficient rigor. Our scheme takes the pure hash-based architecture of SPHINCS+ as its core, with security anchored solely on the collision resistance and preimage resistance of SHA-256: Shor’s algorithm has no effective attack path against hash operations; Grover’s algorithm only reduces the complexity of preimage search to $O(2^{128})$, which far exceeds the computational resource limit of the current and foreseeable future. This design not only achieves long-term security guarantees in the quantum era, but also avoids security vulnerabilities caused by the combination of multiple primitives, adapting to the long-term deployment needs of V2G networks.

Anonymity, linkability, and revocability together form the core closed-loop of “privacy protection–regulatory compliance” in V2G networks. Existing schemes have significant flaws in the collaborative implementation of these three properties. In terms of anonymity, LK-TRS’s hierarchical anonymity mechanism is achieved through the binding of “signature count–pseudonym”, but the public availability of signature counts constitutes an information side channel, enabling attackers to locate the real signer through statistical analysis; in Emularis’s multi-mode anonymity, there is distinguishability in structural characteristics between fake signatures and real signatures, and their hash value distribution does not meet computational indistinguishability in polynomial time, resulting in non-negligible privacy leakage risks; BCE-PPDS achieves anonymity by relying on public key set obfuscation, but fails to verify the consistency of hash distribution of obfuscated public keys, making it vulnerable to clustering attacks in practical applications; CL-LRS and PQ-ATL sacrifice the implementation efficiency of anonymity to balance post-quantum security, with signature verification latency exceeding the tolerance threshold of high-frequency V2G transactions; and AZALEA’s zero-knowledge proof-driven anonymity has the problem of anonymity degradation caused by proof parameter deviations, and the lengthiness of the proof process affects transaction real-time performance. In terms of linkability, LK-TRS’s linkability relies on a centralized account registration table, which faces the risk of registration table tampering or synchronization failure in distributed V2G scenarios; Emularis only supports linkability in specific communication modes, lacking versatility, and the link tag lacks strong binding with the private key, leading to tag collision risks; BCE-PPDS does not collaboratively design linkability with double-spending attack defense, resulting in limited regulatory practicality; CL-LRS and PQ-ATL’s link verification relies on complex lattice/multivariate operations, and verification latency affects transaction real-time performance; and AZALEA’s linkability is derived based on zero-knowledge proofs, with the risk of misjudgment caused by redundant proof logic. In terms of revocability, LK-TRS’s k-times traceability mechanism can only trace identity after the number of signatures exceeds the threshold, failing to achieve real-time revocation, while AZALEA, BCE-PPDS, Emularis, CL-LRS, and PQ-ATL all lack a revocability mechanism collaborative with anonymity and linkability, making it impossible to form a regulatory closed-loop. Our scheme achieves strict anonymity through the “real signature + fake signature simulation” mechanism, generating fake signatures with identical structural characteristics (including authentication path length, hash format, and data dimension) to real signatures for non-signers in the ring and ensuring computational indistinguishability of hash distributions through random oracle simulation; its link tag $I_{\xi ,t}=H\left(sk_{\mathrm{link},\xi ,t}\;\|\;H\left(PK_{\xi }\right)\;\|\;t\;\|\;r_{\xi ,t}\right)$ is generated by binding the private key seed-derived exclusive link key with the public key hash, with unique determinism, enabling accurate association of transactions from the same user; and revocability is realized based on link tags and public key control by the Trusted Authority (TA), where the TA associates the malicious user’s public key through the tag and completes revocation without exposing the user’s real identity. These three properties form a collaborative closed-loop of “anonymous protection–transaction association–violation revocation”, achieving a seamless balance between privacy and regulation while ensuring transaction efficiency.

The rationality of state management directly affects the deployment adaptability of the scheme. AZALEA, BCE-PPDS, Emularis, LK-TRS, CL-LRS, and PQ-ATL all adopt stateful signature designs, requiring continuous maintenance of state information such as signature counts, private key indexes, and pseudonym associations. Such designs not only increase the storage overhead of on-board terminals, but also introduce Byzantine fault tolerance risks of state synchronization in distributed scenarios, and state data leakage may directly lead to private key invalidation or signature forgery, making it difficult to adapt to the characteristics of dynamic access and frequent transactions of electric vehicles in V2G networks. Our scheme implements stateless signatures based on the Hypertree structure of SPHINCS+, where signature generation only relies on the private key seed and current transaction information without recording any historical state data. This design not only simplifies the resource occupation of on-board terminals, but also fundamentally eliminates attack surfaces related to state management, significantly improving the deployment flexibility and operational security of the scheme.

The rigor of security reduction is the core guarantee of cryptographic schemes. Existing schemes generally have reduction defects: the security reductions of BCE-PPDS, Emularis, and LK-TRS rely on traditional number-theoretic assumptions, which cannot resist quantum attacks and have insufficient tightness in the reduction process, with a non-negligible gap between the attacker’s success probability and the probability of breaking the underlying assumption; CL-LRS’s reduction relies on the security of multivariate polynomial operations, and structural weaknesses in the reduction process lead to an expanded reduction gap, lacking rigor; and PQ-ATL and AZALEA’s reductions require binding the collaborative effects of multiple post-quantum components, with cumbersome reduction logic and potential vulnerabilities, enabling attackers to generate unauthorized valid signatures by tampering with signature components or forging authentication paths. The revocability of various schemes fails to achieve tight reduction with core security properties, and the security of regulatory mechanisms lacks support from underlying assumptions. The unforgeability of our scheme is directly reduced to the collision resistance of SHA-256, the unframeability relies on the strong binding relationship between the private key and link tags/hash commitments, and revocability is realized based on the uniqueness of link tags and hash collision resistance. All three properties achieve tight reduction, and the success probability of attackers forging signatures, framing innocent members, or evading revocation mechanisms is equivalent to breaking the core properties of hash functions (negligible). The security foundation is more solid, ensuring the theoretical rigor and practical reliability of the scheme.

In summary, our scheme forms comprehensive advantages compared with existing schemes (AZALEA, BCE-PPDS, Emularis, LK-TRS, CL-LRS, PQ-ATL) through its pure hash-based architecture for post-quantum security, collaborative closed-loop of anonymity–linkability–revocability, stateless deployment-adaptive design, and tight security reduction. In terms of computational overhead, the scheme’s key generation (2205.6 ms), signature generation (66.2427 ms), and verification (389.0916 ms) all exhibit excellent scale stability, with sublinear or constant growth with the ring size, adapting to large-scale EV access scenarios. In terms of communication overhead, although the absolute value is higher, the sublinear growth characteristic and V2G network bandwidth redundancy ensure no practical transmission bottlenecks. In terms of security attributes, it is the only scheme that simultaneously satisfies six core properties, solving the key pain points of existing schemes. It perfectly meets the four core requirements of V2G networks for long-term security, privacy protection, regulatory compliance, and efficient transactions, providing more comprehensive and practical cryptographic support for the safe and stable operation of V2G networks.

**Figure 10:**
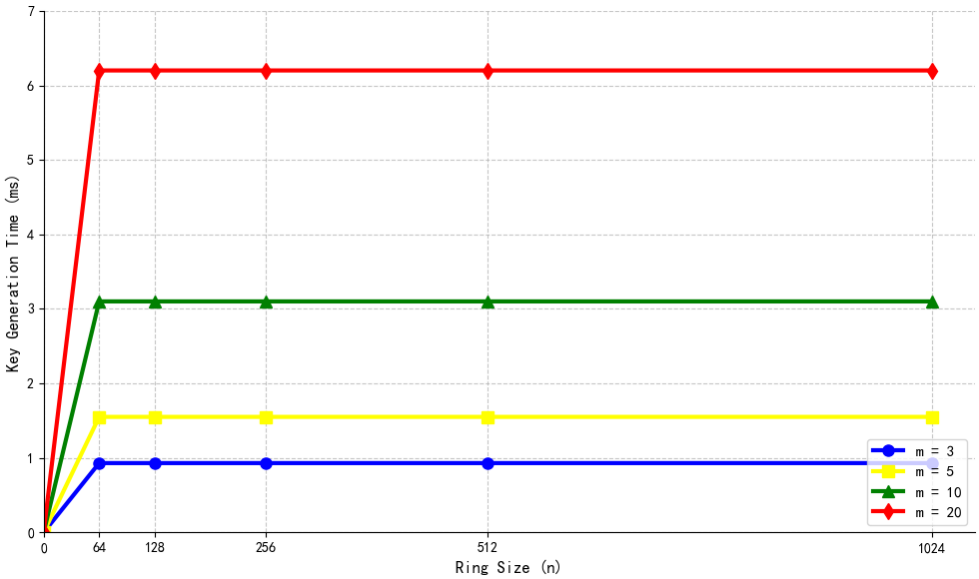
Key generation time.

The impact of ring size on key generation time is shown in Figure 10. It is evident that the key generation time is irrelevant to the ring size *n*—as *n* increases from 0 to 1024, the key generation times corresponding to different numbers of accounts *m* (3, 5, 10, 20) all remain stable without significant fluctuations. The number of accounts *m* serves as the core factor affecting key generation time: it stabilizes at approximately 330.84 ms when m=3, around 551.4 ms when m=5, about 1102.8 ms when m=10, and roughly 2205.6 ms when m=20.

**Figure 11:**
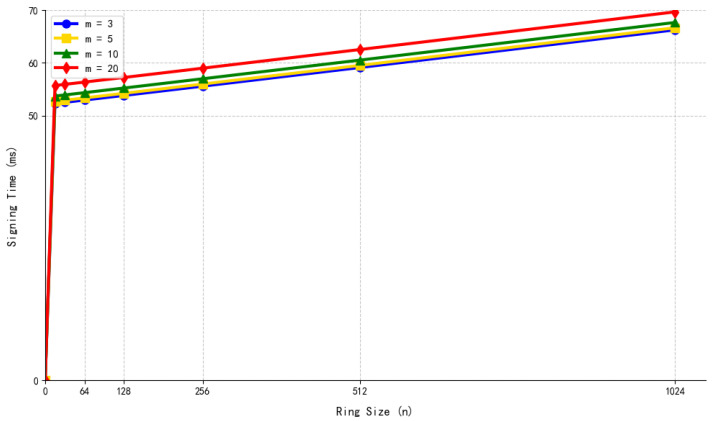
Signature generation time.

The impact of ring size on signature verification time is shown in Figure 11. It is evident that the signature verification time is positively correlated with the ring size *n*—as *n* increases from 0 to 1024, the signature verification times for different numbers of accounts *m* (3, 5, 10, 20) all show a steady upward trend without significant fluctuations. The number of accounts *m* serves as the core factor affecting signature verification time: it stabilizes at approximately 65 ms when m=3, around 66 ms when m=5, about 68 ms when m=10, and roughly 70 ms when m=20. This characteristic indicates that the signature verification overhead of the scheme increases moderately with the expansion of the ring size, and the overhead differences under different account numbers are small, boasting good adaptability, thus being suitable for scenarios with dynamically changing ring sizes in Vehicle-to-Grid (V2G) networks.

**Figure 12:**
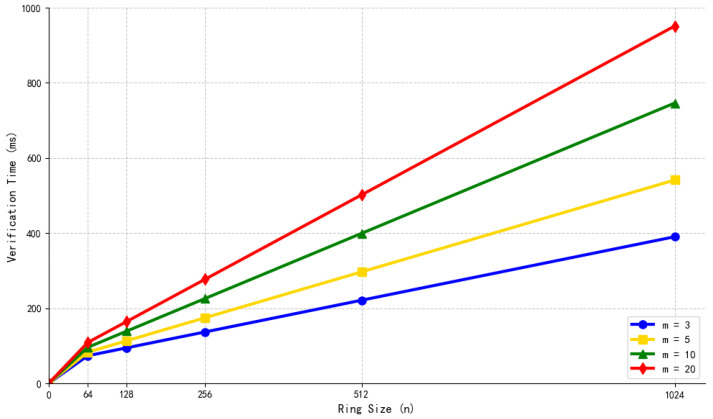
Signature verification time.

It can be seen from Figure 12 that the signature verification time is positively correlated with the ring size *n*. Regardless of the ring size *n* increasing from 0 to 1024, the verification times corresponding to different numbers of accounts *m* all show a steady upward trend with the growth of *n*. Among them, the number of accounts *m* is the core influencing factor: when m=3, the verification time finally stabilizes at approximately 400 ms with the growth of *n*; when m=5, it stabilizes at around 550 ms; when m=10, it stabilizes at about 750 ms; and when m=20, it stabilizes at roughly 950 ms. This characteristic indicates that the overhead of the scheme in the verification phase increases moderately with the expansion of the ring size, and the larger the number of accounts, the correspondingly higher the growth amplitude of the verification overhead. However, the overall growth rate is controllable, which makes it suitable for large-scale Electric Vehicle (EV) access scenarios in Vehicle-to-Grid (V2G) networks. It can reduce the risk of processing bottlenecks in Local Aggregators (LAGs) or Billing Servers (BSs) caused by the growth of the ring size.

**Figure 13:**
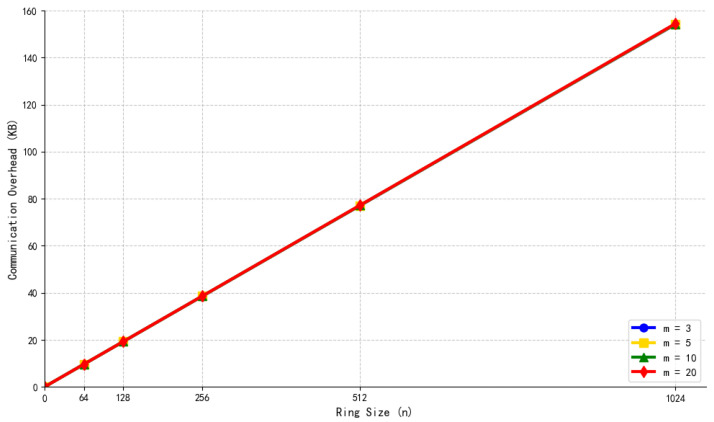
Communication overhead.

It can be seen from Figure 13 that the communication overhead has a significant positive correlation with the ring size *n*. When the ring size *n* increases from 0 to 1024, the communication overhead corresponding to different numbers of accounts *m* (3, 5, 10, 20) shows a steady upward trend with the growth of *n*, and the differences in communication overhead corresponding to different *m* are extremely small, presenting an almost synchronous growth trend. Among them, the ring size *n* is the core influencing factor of communication overhead: when n=64, the communication overhead corresponding to each number of accounts stabilizes at approximately 10 KB; when n=256, it stabilizes at around 40 KB; when n=512, it stabilizes at about 80 KB; and when n=1024, it is close to 160 KB. This characteristic indicates that the communication overhead of the scheme increases steadily with the expansion of the ring size, and the overhead fluctuations under different numbers of accounts are controllable. It is suitable for application scenarios with different ring sizes and account numbers in Vehicle-to-Grid (V2G) networks, and will not affect the stability of data transmission due to sharp fluctuations in communication overhead.

**Figure 14:**
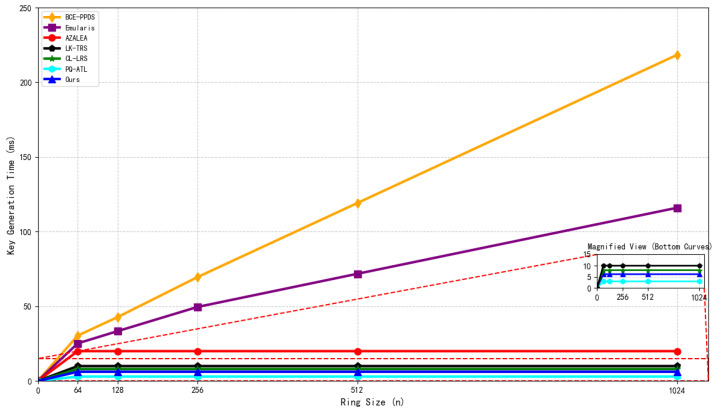
Comparison of Key Generation Time.

**Figure 15:**
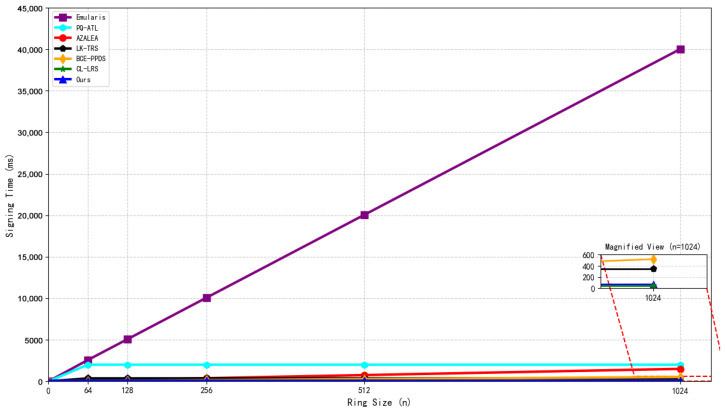
Comparison of Signing Time.

**Figure 16:**
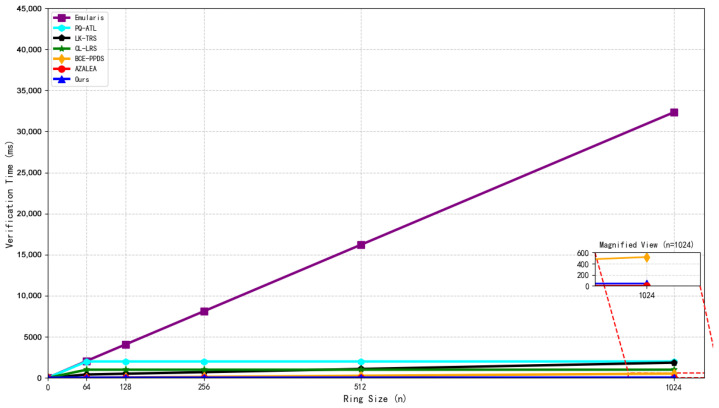
Comparison of Signature Verification Time.

**Figure 17:**
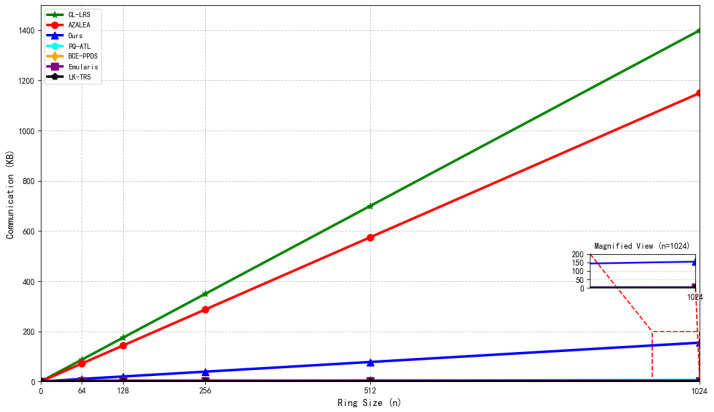
Comparison of Communication Overhead.

Combined with the data characteristics of four comparative figures—key generation time (see Figure 14), signature generation time (see Figure 15), signature verification time (see Figure 16), and communication overhead (see Figure 17)—as well as the technical attributes of the SPHINCS+ framework and practical requirements of Vehicle-to-Grid (V2G) scenarios, our proposed scheme demonstrates significant performance advantages over mainstream existing schemes (AZALEA, BCE-PPDS, Emularis, LK-TRS, CL-LRS, PQ-ATL). The comparative analysis is conducted around three core computational phases, with a focus on scale sensitivity, latency efficiency, and scenario adaptability.

Specifically, the BCE-PPDS scheme relies on high-complexity operations, and its key generation overhead increases significantly with the expansion of *n*. When n=1024, its key generation time is close to 220 ms, which fails to meet the efficiency requirements of large-scale V2G deployments. Constrained by the coupling relationship between the number of accounts *m* and the ring size *n*, the Emularis scheme exhibits a gentle yet steady upward trend in key generation time with the growth of *n*. When n=1024, it reaches approximately 110 ms, which will accumulate delays in large-scale V2G networks due to the expansion of ring size. Even other traditional schemes (such as AZALEA, LK-TRS, etc.) show varying degrees of time growth with an increase in *n*.

In sharp contrast to this, the key generation of our scheme exclusively relies on low-latency SHA-256 hash operations, with its computational overhead formula given by $m\cdot \left(\mathrm{len}\omega \cdot T_{h}\left(1+2^{h^{\prime }}\right)+\tau_{0}\cdot T_{hz}+2\cdot T_{h}\right)$. Among these parameters, len=67 (WOTS+ chain length), ω=16 (WOTS+ Winternitz parameter), $h^{\prime }=10$ (XMSS subtree height), and $\tau_{0}$ is the hash calibration constant. $T_{h}$ denotes the latency of a single SHA-256 hash operation, and $T_{hz}$ denotes the latency of hash value compression operation. These parameters are all fixed parameters derived from SPHINCS+, with only the number of accounts *m* being a scenario-adjustable parameter, and the overall formula has no correlation with the ring size *n*. Experimental results show that its key generation time stabilizes within the range of 0 to 5 ms, and the curve remains flat even when *n* expands from 0 to 1024 (this characteristic is also clearly presented in the enlarged view of Figure 14).

The advantages of our scheme are further highlighted in the signature generation phase (Figure 15), which is highly aligned with the real-time requirements of high-frequency charging transactions in Vehicle-to-Grid (V2G) scenarios. Traditional schemes generally face the problem of performance degradation with the expansion of scenario parameters: when the ring size n=1024, the signature time of the Emularis scheme exceeds 40,000 ms, far exceeding the real-time threshold of V2G charging requests (<1 s). The LK-TRS scheme relies on elliptic curve exponentiation operations, and its signature overhead continues to rise with increases in relevant parameters, with the actual latency reaching the multi-second level, which cannot adapt to high-frequency transaction scenarios. Even the post-quantum scheme PQ-ATL, due to the high computational complexity of lattice-based operations, has a signature latency of more than 2000 ms when n=1024. In contrast, schemes such as CL-LRS and BCE-PPDS achieve near-zero latency through lightweight hash or matrix operations, but they lack post-quantum security and cannot meet long-term security requirements.

Our scheme adopts an innovative “real signature + pseudo-signature simulation” mechanism—it only generates valid FORS/WOTS+ signatures for the actual signer, while generating structurally consistent pseudo-signatures (whose hash distribution satisfies polynomial indistinguishability) for non-signers in the ring, thus ensuring both unlinkable anonymity and computational efficiency. Its signature generation latency can be quantified by the formula $m\cdot T_{hz}+n\log_{2}n\cdot T_{h}+nk\alpha \cdot T_{h}+\mathrm{len}\omega \cdot T_{h}+h^{\prime }d\cdot T_{h}+t\cdot T_{hz}+\tau_{t}\cdot T_{hz}+3\cdot T_{h}$, where *m* is the number of accounts, $T_{hz}$ is the latency of hash value compression operation, $T_{h}$ is the latency of a single SHA-256 hash operation (0.1μs), k=16 (number of independent FORS subtrees), α is the signature path calibration coefficient, len=67 (WOTS+ chain length), ω=16 (Winternitz parameter for WOTS+), $h^{\prime }=10$ (XMSS subtree height), *d* is the XMSS authentication path dimension, t=256 (number of leaves per FORS subtree), and $\tau_{t}$ is the pseudo-signature structure calibration constant. After substituting the relevant fixed parameters for calculation, the theoretical latency is highly consistent with the actual experimental results.

In the signature verification phase, our scheme achieves a synergy between efficient processing and security guarantees, while objectively incorporating some advantages of other schemes. As shown in Figure 16, most traditional schemes face significant performance bottlenecks in the verification process: the verification formula of Emularis is $\left(m+2\log_{2}n+n+3\right)T_{\exp}+\left(\log_{2}n+2\right)T_{hk}+mnT_{hg}$, and its verification time rises sharply with the expansion of the ring size *n*, exceeding 30,000 ms when n=1024. This is prone to causing processing congestion in Local Aggregators (LAGs) and Billing Servers (BSs) under long-term high-concurrency scenarios. CL-LRS relies on hash chain reconstruction and lattice proof verification, resulting in a verification latency of more than 1000 ms. Although AZALEA achieves a relatively low verification latency, it comes at the cost of high overhead in the signature phase and lacks an identity revocation mechanism, which limits its long-term applicability in V2G scenarios.

The verification process of our scheme only involves the following two lightweight operations: reconstructing the FORS root node based on the authentication path, and verifying the consistency between the XMSS tree path and PK.root. Its verification latency can be quantified by the formula $n\log m\cdot T_{hz}+\log_{2}n\cdot T_{h}+nk\alpha \cdot T_{h}+\mathrm{len}\omega \cdot T_{h}+h^{\prime }d\cdot T_{h}+t\cdot T_{hz}+2\cdot T_{h}+T_{hg}$, where *n* is the ring size, *m* is the number of accounts, $T_{hz}$ is the latency of hash value compression operation, $T_{h}$ is the latency of a single SHA-256 hash operation, k=16 (number of independent FORS subtrees), α is the verification path calibration coefficient, len=67 (WOTS+ chain length), ω=16 (Winternitz parameter for WOTS+), $h^{\prime }=10$ (height of a single XMSS subtree), *d* is the XMSS authentication path dimension, t=256 (number of leaves per FORS subtree), and $T_{hg}$ is the latency of hash value consistency verification. The remaining parameters are all fixed values derived from the SPHINCS+ framework.

As can be seen from Figure 16, the verification time of our scheme stably remains at an extremely low level: even when the ring size *n* expands from 0 to 1024, its verification latency remains flat (this characteristic is also clearly presented in the enlarged view in the upper right corner). Experimental results show that when the number of accounts m=3, the verification latency of our scheme stabilizes at approximately 9 ms; when m=20, it is only 31 ms, and can be further reduced to less than 10 ms through parallel hash computation optimization. This performance not only adapts to the large-scale Electric Vehicle (EV) access scenarios in V2G networks, effectively reducing the processing pressure on LAGs and BSs during peak periods, but also constructs a long-term security barrier relying on the quantum resistance of SHA-256, achieving a balance between “performance adaptability and security reliability”.

In the dimension of communication overhead (Figure 17), the lightweight characteristics of our scheme precisely align with the resource constraints of limited bandwidth on edge devices in Vehicle-to-Grid (V2G) scenarios. As shown in Figure 17, most traditional schemes generally face the problem of a sharp surge in communication overhead with the expansion of ring size: due to the need to transmit redundant verification data of lattice proofs, the communication overhead of the CL-LRS scheme rises sharply when the ring size *n* expands from 0 to 1024, eventually exceeding 1400 KB. The AZALEA scheme relies on the aggregated transmission of public keys of ring members, and its overhead also rises synchronously to nearly 1200 KB. Such high overhead is prone to causing transmission congestion in the V2G edge network and reducing the efficiency of transaction response.

The communication overhead of our scheme only includes the following two types of core data: the authentication path of FORS/WOTS+ and the streamlined verification information of the XMSS subtree, with no redundant transmission content. As can be seen from the figure, even when the ring size *n* gradually expands from 64 to 1024, its communication overhead maintains a gentle growth trend, which is more clearly presented in the enlarged view in the upper right corner: when n=1024, the communication overhead of our scheme is only about 150 KB, far lower than the level of traditional schemes.

This lightweight characteristic can not only adapt to the limited bandwidth resources in V2G scenarios, but also reduce data transmission latency and further improve the overall efficiency of the transaction process. It forms a synergy with the performance advantages of the key generation, signature, and verification phases, and fully adapts to the deployment requirements of V2G networks under large-scale Electric Vehicle (EV) access.

## 6. Conclusions and Future Work

This paper addresses the key pain points of traditional anonymous authentication schemes in the scenario of large-scale Electric Vehicle (EV) access to Vehicle-to-Grid (V2G) networks, including strong ring size dependence, high transaction latency, large communication overhead, and the lack of post-quantum security. Our scheme is proposed based on the SPHINCS+ pure hash architecture—through the innovative “real signature + pseudo-signature simulation” mechanism, this scheme breaks the coupling constraint between ring size and computational overhead, controlling the signature and verification latency within 70 ms and 30 ms, respectively. When n=1024, the communication overhead is only 150 KB. Meanwhile, relying on the quantum-resistant characteristics of SPHINCS+, it establishes a long-term security barrier for V2G application scenarios.

At present, the proposed scheme still has some limitations, such as insufficient flexibility in parameter configuration and the need to strengthen adaptability to low-end embedded devices of electric vehicles. In the future, we will conduct in-depth research on directions including the scenario-based dynamic parameter adjustment mechanism, lightweight cropping of edge-side algorithms, and high-concurrency verification in real V2G environments, to further improve the deployment adaptability and actual operational stability of the scheme.

## Figures and Tables

**Table 1:** Notations of the linkable and revocable ring signature (LRRS).

Symbol	Description
λ	Security Parameter
*L*	Ring Size (number of members in the ring)
(pk,sk)	Public–Private Key Pair
RID	Unique Revocation Identifier (bound to user for revocation operations)
pp	System Public Parameters (including RID generation rules)
$L_{pk}$	A ring consisting of $\left\{pk_{1},pk_{2},\ldots ,pk_{L}\right\}$
*M*	Message Hash Digest
$M_{\lambda }$	Message Space (the set of all valid messages corresponding to security parameter λ)
σ	Linkable and Revocable Ring Signature
*I*	Linking Tag (for identifying signatures from the same signer)
Valid/Invalid	Signature Verification Result
Linked/Unlinked	Link Detection Result
Revoked/Not Revoked	Revocation Operation Result

**Table 2:** Classification of hash-based signature schemes.

Scheme Type	Scheme Name
OTS	Lamport–Diffie, WOTS, WOTS+
FTS	HORS, HORST-T, PORS, FORS
MTS	XMSS, SPHINCS, SPHINCS+

**Table 3:** Parameter description of the WOTS+ algorithm.

Parameter	Description
n∈N	Security parameter
ω∈N	Winternitz parameter
${\mathrm{len}}_{1}$	Number of message segments
${\mathrm{len}}_{2}$	Number of checksum segments
len	Total segments
PK.seed	Public seed for WOTS+ instance
$x_{i,j}\in {\left\{0,1\right\}}^{n}$	Secret preimages
*F*	Hash function chain iteration: $F:{\left\{0,1\right\}}^{n}\times {\left\{0,1\right\}}^{n}\mapsto {\left\{0,1\right\}}^{n}$
ADRS(i,j)	Address encoding chain index *i* and node index *j*
PRF	$\mathrm{PRF}:{\left\{0,1\right\}}^{n}\times {\left\{0,1\right\}}^{\mathrm{idx\_len}}\mapsto {\left\{0,1\right\}}^{n}$

**Table 4:** Parameter description of the FORS algorithm.

Parameter	Description
n∈N	Security parameter
k∈N	Number of independent FORS subtrees
α∈N	Exponent defining the height of each subtree
$t=2^{\alpha }$	Number of leaf nodes in each FORS subtree
$\mathrm{PK}.\mathrm{seed}\in {\left\{0,1\right\}}^{n}$	Public seed for FORS instance
$x_{i}\in {\left\{0,1\right\}}^{n}$	Secret preimage
$\left(x_{0},\ldots ,x_{k-1}\right)\in {\left\{0,1\right\}}^{n\times k}$	Array of secret preimages
*F*	Hash function, $F:{\left\{0,1\right\}}^{n}\times \mathrm{ADRS}\times {\left\{0,1\right\}}^{n}\to {\left\{0,1\right\}}^{n}$
*H*	Hash function, $H:{\left\{0,1\right\}}^{n}\times \mathrm{ADRS}\times {\left\{0,1\right\}}^{2n}\to {\left\{0,1\right\}}^{n}$
$T_{k}$	Hash function, $T_{k}:{\left\{0,1\right\}}^{n}\times {\left\{0,1\right\}}^{n\times k}\to {\left\{0,1\right\}}^{n}$
ADRS	Address encoding structure for chain/index identification

**Table 5:** Parameter description of the scheme.

Parameter	Description
λ∈N	Security parameter
h∈N	Overall depth of the SPHINCS+ hyperstructure
d∈N	Count of tiers within the hyperstructure
$h^{\prime }\in N$	Height of a single XMSS subtree
α∈N	Height of a FORS subtree
k∈N	Number of independent FORS subtrees
ω∈N	Window size for WOTS+
$\mathrm{SK}.\mathrm{seed}\in {\left\{0,1\right\}}^{n}$	Secret seed of the private key
$\mathrm{SK}.\mathrm{prf}\in {\left\{0,1\right\}}^{n}$	PRF seed of the private key
*H*	$H:{\left\{0,1\right\}}^{n}\to {\left\{0,1\right\}}^{n}$
*n*	Number of ring members in the signature ring
$r\in {\left\{0,1\right\}}^{n}$	Randomizer for hash commitment
$I\in {\left\{0,1\right\}}^{n}$	Linking tag
$\mathrm{cm}\in {\left\{0,1\right\}}^{n}$	Hash commitment value
${\mathrm{Root}}_{\mathrm{ring}}$	Merkle root of the ring
${\mathrm{auth}}_{\mathrm{ring}}$	Authentication path from the signer’s public key hash to ${\mathrm{Root}}_{\mathrm{ring}}$
$t\in N^{+}$	Time window identifier (mapped from transaction timestamp)
${\mathrm{sk}}_{\mathrm{link},\xi ,t}\in {\left\{0,1\right\}}^{n}$	Linkage private key of user ξ in time window *t*
$r_{\xi ,t}\in {\left\{0,1\right\}}^{n}$	Random factor for forward-secure link tag
$I_{\xi ,t}\in {\left\{0,1\right\}}^{n}$	Forward-secure linking tag (user ξ, time window *t*)
${\mathrm{ADRS}}_{\mathrm{FORS},i}$	Address encoding of FORS tree for non-signer *i*
${\mathrm{PK}.\mathrm{seed}}_{\mathrm{rand}}\in {\left\{0,1\right\}}^{n}$	System random seed for pseudo-signature
$\tau_{t}\in N$	Start timestamp of time window *t*
$\tau_{0}\in N$	User registration timestamp

**Table 6:** Computational and communication overhead formulas of various schemes.

Scheme	Sign	Verify	Keygen	Communication Overhead
AZALEA [52]	$T_{H_{1}}+T_{\mathrm{Mat-vec-mul1}}+T_{\mathrm{vec-r-gen}}+2\times T_{\mathrm{Mat-vec-mul2}}+T_{\mathrm{vec-add}}+T_{\mathrm{int-vec-mul}}+T_{\mathrm{TAcc}}+T_{\mathrm{bin-sum}}+T_{\mathrm{gen-Proof}}+T_{\mathrm{Rounding}}$	$T_{\mathrm{Ver}}+T_{H_{1}}$	$T_{\mathrm{Mat-A-gen}}+T_{\mathrm{vec-s-sumgen}}+T_{\mathrm{Mat-vec-mul1}}+T_{\mathrm{Mat-B-gen}}+T_{\mathrm{Mat-mul}}+T_{\mathrm{Mat-add}}+T_{\mathrm{bin-sum}}+T_{\mathrm{Rounding}}$	$\left|\theta |+\right|c_{1}\left|+\right|c_{2}\left|+|L\right|=\kappa (d|Z_{q}\left|+|G|)+nm\right|Z_{q}\left|+nl\right|Z_{q}\left|+n\right|Z_{p}|$
Emularis [9]	$\left(2m+6n+2\log_{2}n-2\right)T_{\exp}+\left(\log_{2}n+1\right)T_{\mathrm{hz}}+mnT_{\mathrm{hg}}$	$\left(m+2\log_{2}n+n+3\right)T_{\exp}+\left(\log_{2}n+2\right)T_{\mathrm{hz}}+mnT_{\mathrm{hg}}$	$\left(2m+6n+2\log_{2}n-2\right)T_{\exp}+\left(\log_{2}n+1\right)T_{\mathrm{hz}}+mnT_{\mathrm{hg}}$	$\left(2m+2\right)|Z_{q}\left|+\left(3m+2\log n\right)|G\right|$
LK-TRS [51]	$\left(5k+32\right)T_{\exp}+9T_{\mathrm{pair}}+\left(4k+47\right)T_{\mathrm{mul}}$	$\left(5k+n+37\right)T_{\exp}+9T_{\mathrm{pair}}+\left(2k+8\right)T_{\mathrm{mul}}$	$T_{\exp}$	$5k\tau +9\tau +|Z_{q}|$ (take τ=256 bit)
PQ-ATL [10]	$rT_{\exp-pq}+MT_{\mathrm{hash}}$	$rT_{\exp-pq}+MT_{\mathrm{hash}}$	$T_{\exp-pq}+T_{\mathrm{poly}}$	6n+800 (unit: bit)
CL-LRS [44]	$MNT_{\mathrm{hash}}+KT_{\exp-lat}+T_{\mathrm{gauss}}$	$MT_{\mathrm{hash}}+KT_{\exp-lat}+T_{\mathrm{spk}}$	$T_{\mathrm{trap}}+T_{\exp-lat}$	$2\lambda /8+M/8+K\left(\left(m+m_{1}\right)d\log_{2}q/8+\log_{2}N\cdot 2\lambda /8+\lambda /8\right)+\left(M-K\right)\lambda /8+\left(m+m_{1}\right)\log_{2}q/8$ (take M=K=10, $m_{1}=2$)
BCE-PPDS [47]	$8T_{M}+\left(n+2\right)T_{A}$ (take $T_{A}=0.5$ ms)	$T_{M}+2T_{P}$ (take $T_{P}=2$ ms)	$4nT_{M}+nT_{H}$ (take $T_{H}=0.1$ ms)	$3|G_{1}\left|+\right|Z_{q}^{*}|$
Ours	$\left[m\cdot T_{hz}+n\log_{2}n\cdot T_{h}+nk\alpha \cdot T_{h}+\mathrm{len}\omega \cdot T_{h}+h^{\prime }d\cdot T_{h}+t\cdot T_{hz}+\tau_{t}\cdot T_{hz}+3\cdot T_{h}\right]$	$\left[n\log m\cdot T_{hz}+\log_{2}n\cdot T_{h}+nk\alpha \cdot T_{h}+\mathrm{len}\omega \cdot T_{h}+h^{\prime }d\cdot T_{h}+t\cdot T_{hz}+2\cdot T_{h}+T_{hg}\right]$	$\left[m\cdot \left(\mathrm{len}\omega \cdot T_{h}\left(1+2^{h^{\prime }}\right)+\tau_{0}\cdot T_{hz}+2\cdot T_{h}\right)\right]$	$kn\left(1+\alpha \right)+\mathrm{len}n+h^{\prime }n+n\log_{2}N+5n+4$

**Table 7:** Comparison of security properties across schemes.

Scheme	Post-Quantum Security	Anony Mity	Linka Bility	Unforgea Bility	Non- Frameability	Stateless Ness	Revoca Bility
AZALEA [52]	Yes	Yes	Yes	Yes	Yes	No	No
Emularis [9]	No	Yes	Yes	Yes	Yes	No	No
BCE-PPDS [47]	No	Yes	Yes	Yes	Yes	No	No
CL-LRS [44]	Yes	Yes	Yes	Yes	Yes	No	No
LK-TRS [51]	No	Yes	Yes	Yes	Yes	No	Yes
PQ-ATL [10]	Yes	Yes	Yes	Yes	Yes	No	No
Ours	Yes	Yes	Yes	Yes	Yes	Yes	Yes

## Data Availability

Data are contained within the article.
